# Cross-reactivity, antivenomics, and neutralization of toxic activities of *Lachesis* venoms by polyspecific and monospecific antivenoms

**DOI:** 10.1371/journal.pntd.0005793

**Published:** 2017-08-07

**Authors:** Marvin Madrigal, Davinia Pla, Libia Sanz, Elexandra Barboza, Cynthia Arroyo-Portilla, Carlos Corrêa-Netto, José María Gutiérrez, Alberto Alape-Girón, Marietta Flores-Díaz, Juan J. Calvete

**Affiliations:** 1 Instituto Clodomiro Picado, Facultad de Microbiología, Universidad de Costa Rica, San José, Costa Rica; 2 Departamento de Bioquímica, Facultad de Medicina, Universidad de Costa Rica, San José, Costa Rica; 3 Laboratorio de Venómica Estructural y Funcional, Instituto de Biomedicina de Valencia, C.S.I.C., Valencia, Spain; 4 Instituto Vital Brazil, Niterói, Rio de Janeiro, Brazil; Liverpool School of Tropical Medicine, UNITED KINGDOM

## Abstract

**Background:**

*Bothrops*, *Crotalus* and *Lachesis* represent the most medically relevant genera of pitvipers in Central and South America. Similarity in venom phenotype and physiopathological profile of envenomings caused by the four nominal *Lachesis* species led us to hypothesize that an antivenom prepared against venom from any of them may exhibit paraspecificity against all the other congeneric taxa.

**Methods:**

To assess this hypothesis, in this work we have applied antivenomics and immunochemical methods to investigate the immunoreactivity of three monovalent antivenoms and two polyvalent antivenoms towards the venoms from different geographic populations of three different *Lachesis* species. The ability of the antivenoms to neutralize the proteolytic, hemorrhagic, coagulant, and lethal activities of the seven *Lachesis* venoms was also investigated.

**Results:**

A conspicuous pattern of immunorecognition and cross-neutralization for all effects was evident by the polyspecific antivenoms, indicating large immunoreactive epitope conservation across the genus during more than 10 million years since the Central and South American bushmasters diverged.

**Conclusions:**

Despite the broad geographic distribution of *Lachesis*, antivenoms against venoms of different species are effective in the neutralization of congeneric venoms not used in the immunization mixture, indicating that they can be used equivalently for the clinical treatment of any lachesic envenoming.

**General significance:**

This study demonstrates that antivenoms raised against venom of different *Lachesis* species are indistinctly effective in the neutralization of congeneric venoms not used in the immunization mixture, indicating that antivenoms against conspecific venoms may be used equivalently for the clinical treatment of envenomings caused by any bushmaster species.

## Author summary

Snakebite envenoming is a neglected public health problem in many developing countries and antivenom administration constitutes the mainstay in the treatment of such envenomings. Therapeutic antivenoms contain animal-derived antibodies against venom toxins and are produced by immunizing animals with the venom from one or several snake species from a defined geographical area. Defining the geographic boundaries of the efficiency of an antivenom therefore has implications for its rational and efficient use. In Central and South America most accidents are caused by pitvipers of the genus *Bothrops*, *Crotalus* and *Lachesis*. There are four Lachesis species distributed in a variety of habitats ranging from the Caribbean coast of Central America to the Atlantic rainforest of Brazil. *Lachesis* species cause severe envenomings in humans due to the toxicity of their venoms and also to the large amount of venom they inject into their victims. In this work we investigate the capability of several antivenoms to neutralize the toxic activities of a panel of *Lachesis* venoms. The results demonstrate that antivenoms raised by immunizing horses with the venoms of different *Lachesis* species are effective at neutralizing congeneric venoms not used in the immunization, indicating that they could be used equivalently for the clinical treatment of any lachesic envenoming.

## Introduction

Snakebite envenoming is a public health issue and a neglected disease in many tropical and sub-tropical regions of Africa, Asia, Latin America and Oceania, especially affecting the most impoverished and geopolitically disadvantaged rural communities [1–4]. Between 1.2 and 5.5 million people are victims of snakebites every year, leading to 95.000–125.000 deaths and leaving more than 400.000 people with permanent physical and psychological sequelae [4–7]. In Central and South America most accidents are caused by pitvipers of the Viperidae family, subfamily Crotalinae, with *Bothrops*, *Crotalus* and *Lachesis* being the most medically relevant genera.

Genus *Lachesis* comprises the longest pitvipers in the world, with adults ranging in length from 2 to 2.5 m. The four nominal species of this genus, *L*. *stenophrys* (Central American bushmaster), *L*. *melanocephala* (Black-headed bushmaster), *L*. *acrochorda* (Chocoan busmaster) and *L*. *muta* (South American bushmaster) inhabit remote forested areas of Central and South America, and on the island of Trinidad [8–10]. Central and South American populations of *Lachesis* diverged around 18.0–6.5 Mya, with a later split between *L*. *melanocephala* and *L*. *stenophrys* taking place 11–4 Mya, while differentiation of South American lineages occurred 800.000 to 300.000 years ago [8]. *L*. *stenophrys* is distributed through the Caribbean coast of Nicaragua, Costa Rica and Panama; *L*. *melanocephala* is found in the Pacific versant of southwestern Costa Rica, and the extreme western regions of Panama; *L*. *acrochorda* inhabits both the Atlantic and Pacific versants of western Panama and into northwestern Colombia, on the Atlantic coast, where it extends southward into the Cauca and Magdalena rivers valleys, and along the Pacific versant of Colombia into northwestern Ecuador. *L*. *muta* is the most widely distributed species of the genus, including the equatorial forest east of the Andes, from Colombia, eastern Ecuador, Peru, northern Bolivia, eastern and southern Venezuela to Guyana, Surinam, French Guiana and most of northern Brazil [10]. Two subspecies of *L*. *muta* are reported: *L*. *m*. *muta* and *L*. *m*. *rhombeata*, with an exclusive distribution of the latter subspecies in the Atlantic forest of eastern-center of Brazil. Based on morphology, some authors consider that there are populations of *L*. *m*. *rhombeata* in the Amazonia basin [11].

Human bites by *Lachesis* species are not frequent but when occur cause severe envenoming due to large amount of venom (200–411 mg) injected into the victim and also owing to its toxicity in humans, as reported for snakebites in Brazil, Colombia and Costa Rica [11–21]. Common local effects include agonizing burning-throbbing pain, mild hemorrhage, edema, and blister formation. These signs and symptoms are accompanied by systemic alterations, such as hemorrhage, coagulopathy, cardiovascular collapse, and by the so-called “*Lachesis* syndrome”, an alteration of the autonomic nervous system which manifests with profuse sweating, abdominal colic, nausea, recurrent vomiting, watery diarrhea, diastolic and systolic hypotension, and sinus bradycardia, together with sensorial disorders (uncoordinated march, lapses of unconsciousness) and serious hemodynamic alterations within 15–20 min after a bite [12–19, 22].

Comprehensive transcriptomic and proteomic studies across *Lachesis* [23–26] have revealed remarkably similar venom phenotypes comprising seven or eight toxin families, including bradykinin-potentiating/C-type natriuretic peptide (BPPs/C-NP), Zn^2+^-dependent snake venom (SV) metalloproteinase (SVMP), serine protease (SVSP), phospholipase A_2_ (PLA_2_), L-amino acid oxidase (LAOs), C-type lectin-like (CTL), and in venoms of the South American species, also cysteine-rich secretory protein (CRISP). Ontogenetic changes in the toxin composition of *L*. *stenophrys* venom result in the net shift from a BPPs/C-NP-rich and SVSP-rich venom in newborns and 2-years-old juveniles to a (PI>PIII) SVMP-rich venom in adults [24].

The high conservation of the overall composition of Central and South American bushmaster venoms and their qualitatively similar pathophysiological profile observed in clinical settings [14,17,27–29], suggested that antivenoms generated against any conspecific *Lachesis* venom may exhibit paraspecific protection against the toxic activities of all other *Lachesis* species [24,25]. The aim of the present work was to assess this hypothesis. To this end, we carried out a comparative study of the cross-reactivity, neutralization of toxic activities and immunoaffinity antivenomic profiles towards a panel of *Lachesis* venoms of two commercial polyspecific antivenoms (BCL), manufactured at Instituto Clodomiro Picado, Costa Rica, against a mixture of *L*. *stenophrys*, *Bothrops asper*, and *Crotalus simus* venoms, and antivenom produced at Instituto Vital Brazil, Brazil, against venoms from *L*. *m*. *rhombeata* and five bothropic species (BL), and experimental monospecific antivenoms AL, AB, and AC, generated, respectively, against venoms of adult Costa Rican *L*. *stenophrys*, *B*. *asper*, and *C*. *simus*.

## Methods

### Ethics statement

All the procedures involving the use of animals in this study were approved by the Institutional Committee for the Care and Use of Laboratory Animals (CICUA) of Universidad de Costa Rica (approval number CICUA 028–13), and meet the Animal Research Reporting *in vivo* Experiments (ARRIVE) guidelines, and the International Guiding Principles for Biomedical Research Involving Animals of the Council of International Organizations of Medical Sciences (CIOMS).

### Snake venoms and antivenoms

*Lachesis* venoms were obtained from different geographic areas of Central and South America. Venom from *L*. *stenophrys* (Central American bushmaster) was pooled from more than 25 adult (>5 years old) snakes maintained in the herpetarium of Instituto Clodomiro Picado (ICP, San José, Costa Rica). Venom from *L*. *melanocephala* (black-headed bushmaster) was pooled from two adult specimens maintained at Instituto Nacional de Biodiversidad (San José, Costa Rica). *L*. *muta muta* (South American bushmaster) venoms pooled from adult specimens from Colombia, Peru, and Cascalheria and Tucurui regions of Brazil were kindly provided by Dr. María de Fatima D. Furtado (Instituto Butantan, São Paulo, Brazil). Samples of *L*. *muta rhombeata* (Atlantic forest bushmaster) venom pooled from adult specimens were a generous gift from Dr. María de Fatima D. Furtado of Instituto Butantan and from Instituto Vital Brazil (IVB, Niterói, Rio de Janeiro, Brazil). All venoms were lyophilized and stored at -20°C until used.

Commercial polyspecific BCL antivenom (batch 4800611POLQ) was manufactured by the Industrial Division of Instituto Clodomiro Picado (San José, Costa Rica) from the plasma of horses hyperimmunized with a mixture of venoms of Costa Rican *Bothrops asper*, *Crotalus simus* and *Lachesis stenophrys* [30, 31], and consists of whole IgGs purified by caprylic acid fractionation [32]. BL antivenom (batch 125901) from Instituto Vital Brazil (Niterói, RJ, Brazil) was produced in horses hyperimmunized with a mixture of venoms from *L*. *m*. *rhombeata* and a mixture of five bothropic species, *B*. *jararaca* (50%), *B*. *jararacussu* (12.5%), *B*. *moojeni* (12.5%), *B*. *alternatus* (12.5%) and *B*. *neuwiedi* (12.5%), and consists of purified F(ab')_2_ fragments generated by digestion with pepsin of ammonium sulfate-precipitated IgG molecules [33]. Experimental monospecific AB, AC, and AL antivenoms were prepared by the Industrial Division of Instituto Clodomiro Picado from plasma of horses subjected to a single round of immunization with venoms of Costa Rican adult *B*. *asper* (from the Pacific and Caribbean versants of Costa Rica), adult *C*. *simus*, and adult *L*. *stenophrys*, respectively, as described [33]. These monospecific antivenoms are also whole IgG preparations prepared by caprylic acid precipitation [33]. BCL and BL antivenoms are used therapeutically in the clinical management of *Lachesis* envenomings in Central America and Brazil, respectively. Monospecific AC, AB and AL antivenoms were developed for experimental use. For *in vitro* and *in vivo* assays the protein concentration of antivenoms was adjusted to 50 mg/mL.

### ELISA (Enzyme-linked immunosorbent assays)

96-well plates (Dynatech Immulon, Alexandria, VA) were coated overnight at 4°C with *Lachesis* venoms (0.5 μM/well) in 0.1 M Tris, 0.15 M NaCl, pH 9.0 buffer. The plates were blocked for 1h with 2% bovine serum albumin (BSA) in 20 mM phosphate, 135 mM NaCl, pH 7.4 (PBS) at room temperature. Purified antivenom immunoglobulins were serially diluted by a factor of 3 (starting from a dilution of 1/500) in PBS containing 1% BSA, and added to the wells for 1 h at room temperature. The plates were washed four times with washing buffer (50 mM Tris, 150 mM NaCl, 20 μM ZnCl_2_, 1 mM MgCl_2,_ pH 7.4), and anti-horse IgG-phosphatase-conjugate (Sigma, St. Louis, MO, USA), diluted 1:20,000 with PBS containing 1% BSA, was added and incubated for 1 h at room temperature. The plates were washed and developed with *p*-nitrophenylphosphate in diethanolamine buffer (1 mM MgCl_2_, 90 mM diethanolamine, pH 9.8). Absorbance at 405 nm was recorded after 90 min using a microplate reader (Multiskan Labsystems Ltd., Helsinki, Finland).

### Two-dimensional electrophoresis

*L*. *stenophrys* venom proteins were separated by two-dimensional electrophoresis (2DE) using an Ettan IPGphor III instrument (GE Healthcare Bio-Sciences AB, Uppsala, Sweden). For isoelectric focusing, 300–350 μg of total venom proteins in 200 μL DeStreak Rehydration Solution (GE Healthcare Bio-Sciences AB, Uppsala, Sweden) including 10 mM DTT and 0.5% IPG buffer pH 3–10 NL (GE Healthcare Bio-Sciences AB, Uppsala, Sweden) were loaded on a 11 cm IPG strip, pH 3–10 (GE Healthcare Bio-Sciences AB, Uppsala, Sweden) and then focused using the following electrophoretic conditions: 500 V for 30 min, 1000 V for 30 min and 5000 V for 80 min. After isoelectric focusing, SDS-PAGE was performed under reducing conditions in 4–15% Criterion TGX precast 11 cm gels (Bio-Rad, USA). An unstained protein molecular weight marker (Fermentas) was included in the analysis. Gels were stained using Bio-Safe Coomassie Stain (Bio-Rad, USA) or PlusOne Silver Staining Kit (GE Healthcare AB, Uppsala, Sweden) following the manufacturer´s instructions, and images were taken with Chemidoc XRS imaging system (BioRad, USA). Spot identification was done using the collaborative bioimage informatics platform Icy [34] and quantified as relative density percentage using ImageJ software [35].

### Western blot analysis

2DE gels of 350 μg *L*. *stenophrys* venom proteins were transferred to polyvinylidene fluoride (PVDF) membranes at 50 mA in a Criterion Blotter instrument (Bio-Rad, USA) overnight. To assess transfer efficiency, PVDF membranes were previsualized by reversible Ponceau-S Red staining. Unoccupied membrane protein-binding sites were blocked with 2% casein in TBS-T (Tris-buffered saline with Tween 20, pH 7.6) for 30 min at room temperature, and the membranes were incubated for 1 h with 1/1000 dilution of antivenoms in TBS-T containing 1% casein. After five washing steps (5 min each) with TBS-T, the membranes were incubated for 1 h at room temperature with rabbit anti-horse IgG-peroxidase conjugate (1:15000 dilution; Sigma-Aldrich, St. Louis, USA). Purified antibodies from non-immunized horses were used as control. After washing off unbound secondary antibodies, the immunoreactive spots were visualized using a chemiluminescence substrate (Invitrogen, USA). Images were taken with Chemidoc XRS imaging system (BioRad, USA) and protein spots of interest were analyzed using ImageJ software.

### Protein identification by MALDI-TOF-TOF MS

2DE protein spots were excised and subjected to reduction (10 mM dithiothreitol), alkylation (50 mM iodoacetamide), and overnight in-gel digestion with sequencing grade trypsin (Sigma), in 50 mM ammonium bicarbonate at 37°C. Tryptic peptide digests were extracted in 50% acetonitrile containing 1% trifluoroacetic acid (TFA), and analyzed by MALDI-TOF-TOF MS using an AB4800-Plus Proteomics Analyzer (Applied Biosystems). To this end, tryptic digests were mixed with an equal volume of α-cyano-hydroxycinnamic acid saturated in 50% acetonitrile, 0.1% TFA, and 1 μL spotted onto an Opti-TOF 384-well plate, dried, and analyzed in positive reflector mode. TOF MS spectra were acquired using 500 shots at a laser intensity of 3000. TOF/TOF fragmentation spectra were acquired (500 shots at a laser intensity of 3900) for the ten most intense precursor ions. External calibration in each run was performed with CalMix standards (ABSciex) spotted onto the same plate. Fragmentation spectra were searched against the UniProt/SwissProt database (taxonomy: Serpentes) using the ProteinPilot v.4 and the Paragon algorithm (ABSciex) at ≥95% confidence, or manually interpreted and the deduced sequences BLASTed against the NCBI (http://blast.ncbi.nlm.nih.gov) non-redundant database for protein class assignment by similarity.

### Antivenomics

The immunoreactivity of poly- and monospecific antivenoms towards the different *Lachesis* venoms was assessed using a second-generation antivenomics approach [36]. To prepare the antivenom affinity column, 200 μL of NHS-activated Sepharose 4 Fast Flow (GE Healthcare Bio-Sciences AB, Uppsala, Sweden) matrix was packed in a Pierce centrifuge column and washed with 15 matrix volumes of cold 1 mM HCl followed by two matrix volumes of 0.2 M NaHCO_3_, 0.5 M NaCl, pH 8.3 (coupling buffer) to adjust the pH of the column to 7.0–8.0. Antivenoms were dialysed against MilliQ water, lyophilised, and reconstituted in coupling buffer. The concentration of the antivenom stock solutions was determined spectrophotometrically using an extinction coefficient of 1.36 for a 1 mg/mL concentration of Fab at 280 nm using a 1 cm light pathlength cuvette. Twenty milligrams of polyspecific BCL antivenom, 15 mg of BL antivenom, and 35–50 mg of monospecific AC, AB and AL antivenoms were dissolved in a half matrix volume of coupling buffer and incubated with the matrix for 4 h at room temperature. Antivenom coupling yield was estimated measuring the non-bound antivenom by quantitative band densitometry of SDS-PAGE (MetaMorph software, MDS Analytical Technologies) using as standard for the linear range the pre-coupled antivenom. After the coupling, any remaining active groups were blocked with 200 μL of 0.1 M Tris–HCl, pH 8.0 at 4°C overnight using an orbital shaker. The affinity column was washed alternately at high and low pH, with three volumes of 0.1 M acetate buffer, 0.5 M NaCl, pH 4.0–5.0 and three volumes of 0.1 M Tris–HCl buffer, pH 8.5. This treatment was repeated six times and the column was equilibrated in binding buffer (20 mM phosphate, 135 mM NaCl, pH 7.4, PBS). For the immunoaffinity assay, 200 μg of venoms from *L*. *stenophrys* (Costa Rica), *L*. *melanocephala* (Costa Rica), *L*. *m*. *rhombeata* (Recife, Brazil), and *L*. *m*. *muta* from Colombia, Peru and Brazil (Tucurui and Cascalheira regions), dissolved in 1⁄2 matrix volume of PBS, were loaded and incubated for 1 h at room temperature with the affinity matrix, followed by incubation in an orbital shaker overnight at 4°C. As specificity controls, 200 μL of Sepharose 4 Fast Flow matrix, without or with 8.5 mg of immobilized pre-immune IgGs, were incubated with venom and developed in parallel to the immunoaffinity columns. Non-retained fractions were collected with 5 matrix volumes of PBS, and the immunocaptured proteins were eluted with 5 matrix volumes of elution buffer (0.1 M glycine-HCl, pH 2.0) and neutralised with 150 μL 1 M Tris-HCl, pH 9.0. The non-retained and the immunocaptured venom fractions were lyophilized, reconstituted in 40μl of MilliQ water, and fractionated by reverse-phase HPLC using a Discovery BIO Wide Pore C_18_ (15 cm x 2.1 mm, 3 μm particle size, 300 Å pore size) column using an Agilent LC 1100 High Pressure Gradient System equipped with a DAD detector and micro-auto sampler. The column was developed at a flow rate of 0.4 mL/min and proteins eluted with a linear gradient of 0.1% TFA in MilliQ water (solution A) and 0.1% TFA in acetonitrile (solution B): isocratic at 5% solution B for 1 min, followed by 5–25% solution B for 5 min, 25–45% solution B for 35 min, and 45–70% solution B for 5 min. Protein was detected at 215 nm with a reference wavelength of 400 nm.

### Neutralization of venom activities

Polyspecific and monospecific antivenoms were assessed for their ability to neutralize the lethal, hemorrhagic, coagulant and proteolytic activities of venoms. The protein concentration of all antivenoms was adjusted to 50 mg/mL, as determined using a NanoDrop 2000 (Thermo Fischer Scientific, DE, USA). For the neutralization assays, a fixed dose of venom (“challenge dose”), dissolved in PBS, was incubated with various dilutions of antivenom. Controls including venom solutions incubated with PBS instead of antivenom were used. The venom/antivenom mixtures and controls were incubated for 30 min at 37°C and then tested in the experimental systems described below and detailed in previous publications [37,38]. Neutralizing ability was expressed as Median Effective Dose (ED_50_), defined as the μL antivenom/mg venom ratio in which the activity of venom was reduced by 50% [39]. In the case of coagulant activity, neutralization was expressed as Effective Dose (ED), defined as the antivenom/venom ratio in which the clotting time of plasma was prolonged three times when compared with clotting time of plasma incubated with venom alone [40].

All the *in vivo* experiments were performed in CD-1 mice, and the protocols were approved by the Institutional Committee for the Care and Use of Laboratory Animals (CICUA) of the University of Costa Rica. Lethality was assessed by the intraperitoneal route in 16–18 g mice and a challenge dose corresponded to 3 Median Lethal Doses (LD_50_) was used for the neutralization tests [37]. An arbitrary level of 500 μL antivenom/mg venom was selected to evaluate the efficacy of antivenoms for neutralizing lethality. Only this antivenom/venom ratio was used owing to the scarcity of some venoms and also for reducing the number of mice used. Death of mice was recorded at 48 h. Hemorrhagic activity was evaluated by using the rodent skin test using 18–20 g mice and a challenge venom dose corresponding to 10 Minimum Hemorrhagic Doses (MHD) [41]. Coagulant activity was assessed on citrated human plasma and the challenge dose used was 2 Minimum Coagulant Doses (MCD) [40]. Proteolytic activity was determined using azocasein (Sigma, USA) as substrate, as described by Gutiérrez et al. [42]. For neutralization tests, a challenge dose was selected, corresponding to the amount of venom that induced a change in absorbance of 0.75 at 450 nm. A summary of reference venom activities (Median Lethal Dose, Minimum Hemorrhagic Dose, Minimum Coagulant Dose and challenge dose for proteolytic activity) of *Lachesis* venoms are listed in Table 1.

**Table 1:** Reference doses of *Lachesis* venoms considered in the study.

Venom	LD_50_ [μg]	MHD [μg]	MCD [μg]	Challenge dose (proteolysis) [μg]
***L*. *stenophrys* (CR)**	88.6 (69.8–112.3)^a^	2.10 ± 0.4^b^	6.00 ± 0.9 ^a^	10
***L*. *melanocephala* (CR)**	103^c^	0.70 ± 0.06 ^c^	6.77 ± 0.13	20
***L*. *m*. *muta* (Colombia)**	121.6 (98.7–164.6)^a^	0.66 ± 0.13^a^	8.10 ± 1.6 ^a^	15
***L*. *m*. *muta* (Peru)**	192.4 (170.2–207.4)	0.096 ± 0.02	4.46 ± 0.08	20
***L*. *m*. *muta* (Cascalheira, Br)**	72.7 (56.4–93.9)^a^	0.23 ± 0.03^a^	5.20 ± 0.3^a^	20
***L*. *m*. *muta* (Tucurui, Br)**	107.2 (82.0–140.3)^a^	0.77 ± 0.20^a^	8.70 ± 2.4^a^	10
***L*. *m*. *rhombeata* (Recife, Br)**	122.8 (94.3–160)^a^	0.95 ± 0.12^a^	2.50 ± 0.6^a^	15

LD_50_: Median Lethal Dose; MHD: Minimum Hemorrhagic Dose; MCD: Minimum Coagulant Dose (see text for details).

All challenge doses for proteolytic activity were calculated according to [42].

LD_50_, MHD and MCD for *L*. *muta muta* venom from Peru, and MCD for *L*. *melanocephala* venom are those previously described in [55].

^a^[56]

^b^[57]

^c^[29].

### Statistical analyses

The results of neutralization assays of venom activities were compared by ANOVA, followed by Tukey test for specific comparisons between means of pairs of groups. A p value <0.05 was regarded as statistically significant. For data not following the assumptions of parametric tests, a Kruskal-Wallis test was used, followed by Dunn test. The analysis was performed using the Minitab (v 16.1.0, 2010) statistic program.

## Results and discussion

Venom represents a trophic adaptive trait that plays key roles in the organismal ecology and evolution of advanced snakes [43,44]. The well-documented geographic variability of snake venoms at all taxonomic levels [45] may contribute to the snake's capability to adapt to different ecological niches, but at the same time imposes an added difficulty to the production of antidotes to counteract the toxic activities of snakebite envenomings. The parenteral administration of an effective antivenom constitutes the mainstay in the treatment of snakebite envenomings [46,47]. Defining the geographic boundaries of the efficiency of an antivenom, even against disjunct populations of the same nominal species, has implications for its rational and efficient use. In this regard, unveiling the immunological profile of an antivenom towards the landscape of congeneric venom phenotypic variation provides the necessary knowledge-informed ground for assessing whether it is clinically justified the generation of a new antivenom for a specific geographic region, or if the deployment of an existing antivenom to a new geographical setting can be recommended. This is particularly relevant for widely distributed species, such as those comprising the genus *Lachesis*, which can be found in disjunct habitats ranging from the Caribbean coast of Central America to the Atlantic rainforest of Brazil (see Fig 1 in [24]). The combination of venom neutralization tests and antivenomics constitutes a powerful toolbox for evaluating an antivenom's preclinical efficacy [48–51]. Using this platform we have investigated the capability of two therapeutic polyvalent antivenoms and three experimental monospecific antivenoms to neutralize the hemorrhagic, coagulant, proteolytic and lethal activities of homologous and heterologous *Lachesis* venoms.

### Immunoreactivity profile of Lachesis antivenoms by ELISA and 2DE immunoblotting analysis

Initial assessment of the immunoreactivity of the commercial polyspecific BCL and BL antivenoms, and the experimental monospecific B, C and L antivenoms, against antigens present in the venoms of Costa Rican *L*. *stenophrys* and *L*. *melanocephala*, Brazilian *L*. *m*. *rhombeata* (Recife) and *L*. *m*. *muta* from different geographic locations (Colombia, Peru, and Brazil [Cascalheira and Tucurui]) were done by ELISA and 2DE immunoblotting analysis.

No significant differences were found in the levels of specific antibodies against *Lachesis* venoms present in the BCL antivenom, and the AB and AL antivenoms (S1 Fig). The highest titer corresponded to the binding of BL antivenom to *L*. *stenophrys*, *L*. *m*. *muta* (Colombia), *L*. *m*. *muta* (Cascalheira), and *L*. *m*. *rhombeata* (Recife) venoms, whereas the titer of this antivenom against venoms from *L*. *melanocephala*, *L*. *m*. *muta* (Peru) and *L*. *m*. *muta* (Tucurui) was indistinguishable from that of the BCL antivenom (S1 Fig). Monospecific AC antivenom exhibited the lowest reactivity against the seven *Lachesis* venoms analyzed (S1 Fig).

**Fig 1:**
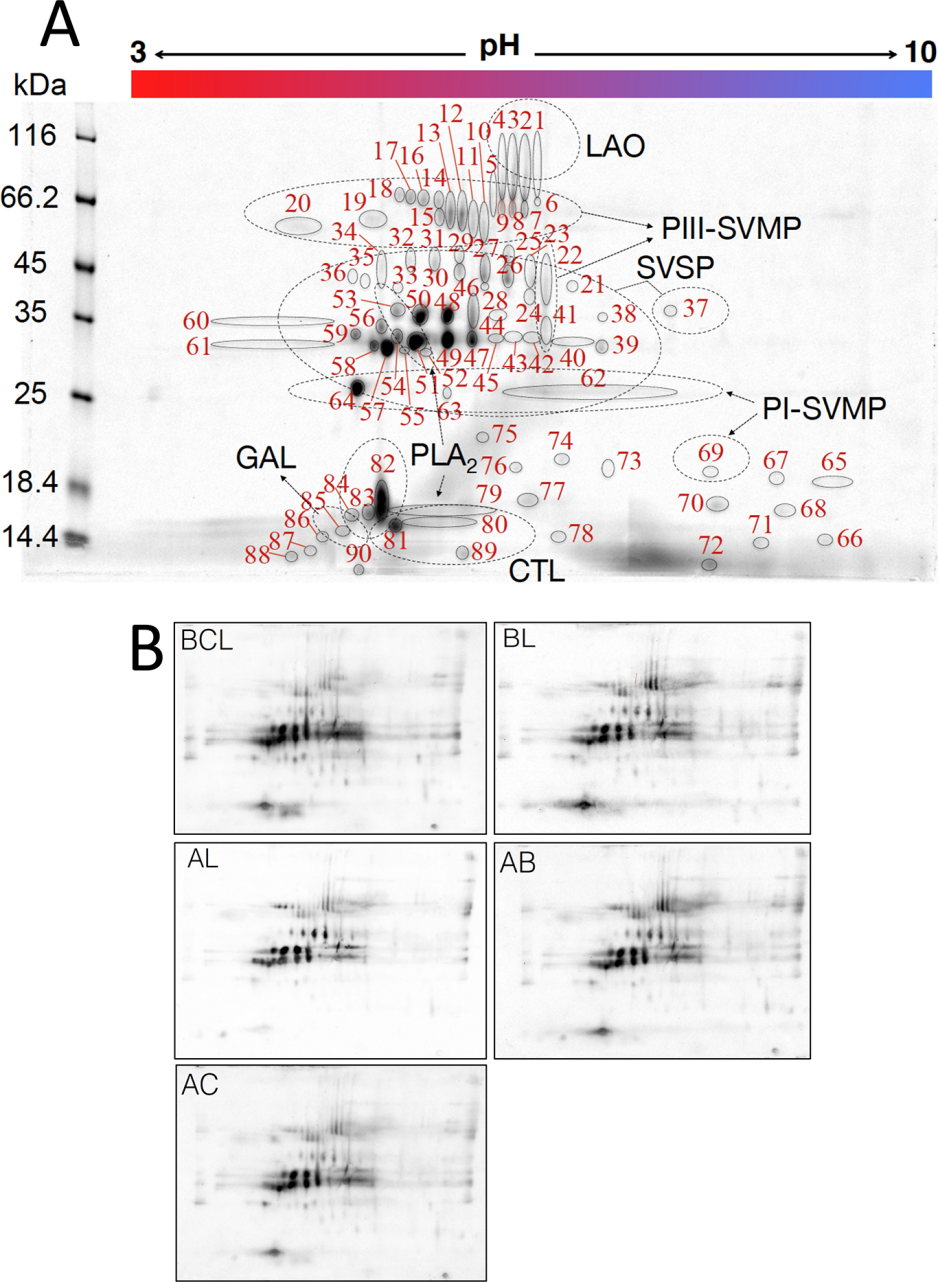
**Panel A.** 2DE protein map of adult *L*. *stenophrys* venom. 350 μg of venom was separated on an 11 cm IPG strip with a pH gradient from 3 to 10 (first dimension) and on a 4–15% SDS-polyacrylamide Criterion TGX gel as second dimension. Proteins were stained with Coomassie Blue. Isoelectric point (IP), apparent Mw, and relative spot intensity were computed using software ImageJ. Protein identification was accomplished by MALDI-TOF-TOF MS. Protein spot features are listed in S1 Table. **Panel B**. Electroblotted 2DE separated venom proteins from adult *L*. *stenophrys* probed against commercial polyspecific BCL antivenom; BL antivenom; and monoespecific AL, AB, and AC antivenoms. Protein identifications by MALDI-TOF/TOF MS are listed in S1 Table (Supplementary Materials).

The spectrum of *L*. *stenophrys* toxins immunorecognized by the poly- and monospecific antivenoms was investigated by 2DE and immunoblot analysis. Fig 1A displays a 2DE reference map and the MALDI-TOF-TOF MS protein assignments are listed in S1 Table. In concordance with ELISA results, Western blot analyses revealed extensive protein spot recognition by all the five antivenoms (Fig 1B and S2 Table), particularly for spots in the range of 25–35 kDa (serine proteinases, SVSPs) and 14–16 kDa (phospholipases A_2_ (PLA_2_) and Galactose-binding lectin). Polyspecific BL and monospecific AB antivenoms showed also strong immunoreactivity towards protein spots of apparent molecular mass 55–80 kDa, which were identified as snake venom metalloproteinases of class PIII (PIII-SVMP) and L-amino acid oxidase (LAO) molecules. However, all the antivenoms showed weak immunostaining of spot 64 containing the major SVMP of class PI (PI-SVMP). Weak immunorecognition of PI-SVMPs has been also reported for other antibothropic antivenoms [52,53].

### Immunoreactivity profile of antivenoms by immunoaffinity-based antivenomics

**Fig 2:**
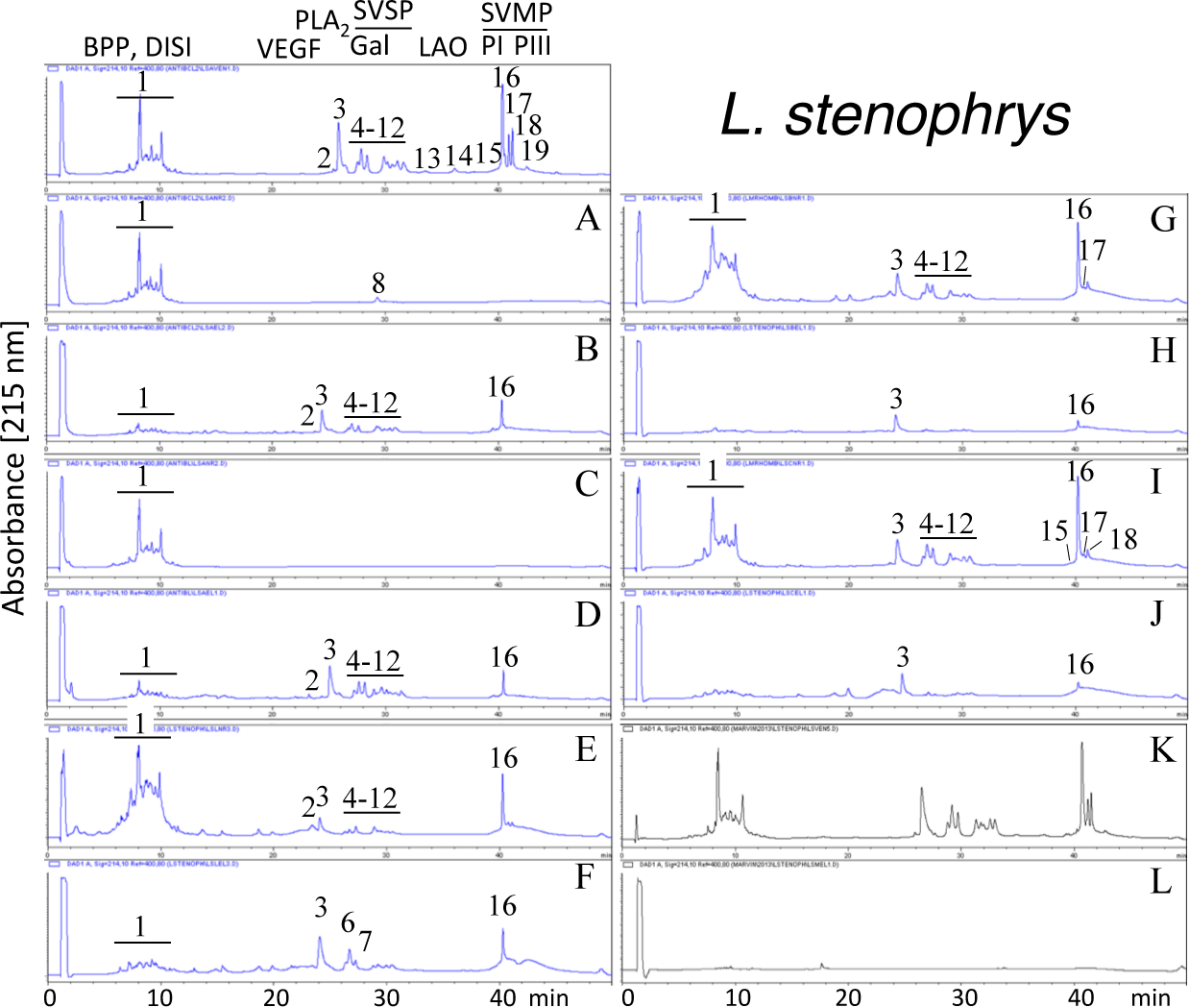
Immunoaffinity chromatography-based antivenomic analysis of the immunoreactivity of polyspecific and monospecific antivenoms towards the venom of *L*. *stenophrys* from Costa Rica. Panels display reverse-phase separations of whole venom components, and non-retained (**A**, **C**, **E**, **G**, and **I**) and the retained (**B**, **D**, **F**, **H**, and **J**) fractions recovered, respectively, from the affinity columns of immobilized BCL, BL, AL, AB, and AC antivenoms. Panels **K** and **L**, non-retained and retained venom fractions by immobilized equine control immunoglobulins. BPP, bradykinin-potentiating peptide; DISI, disintegrin; VEGF, vascular endothelial growth factor; PLA_2_, phospholipase A_2_; Gal, galactose-binding lectin; SVSP, snake venom serine proteinase; LAO, L-amino acid oxidase; PI and PIII, snake venom metalloproteinase (SVMP) of class PI and PIII, respectively.

**Fig 3:**
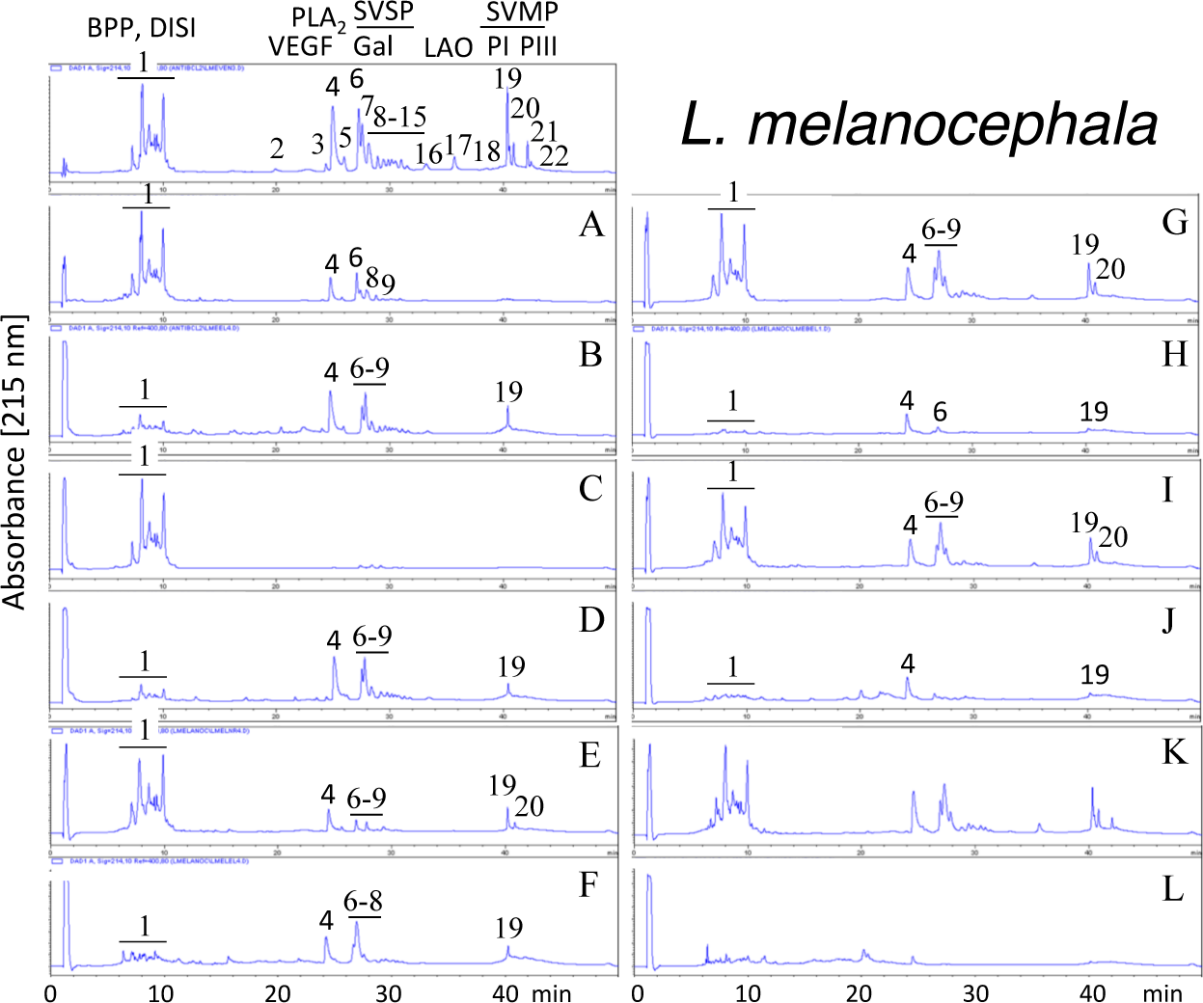
Immunoaffinity chromatography-based antivenomic analysis of the immunoreactivity of polyspecific and monospecific antivenoms towards the venom of *L*. *melanocephala* from Costa Rica. Panels display reverse-phase separations of whole venom components, and non-retained (**A**, **C**, **E**, **G**, and **I**) and the retained (**B**, **D**, **F**, **H**, and **J**) fractions recovered, respectively, from the affinity columns of immobilized BCL, BL, AL, AB, and AC antivenoms. Panels **K** and **L**, non-retained and retained venom fractions by immobilized equine control immunoglobulins. Protein acronyms as in the legend of Fig 2.

**Fig 4:**
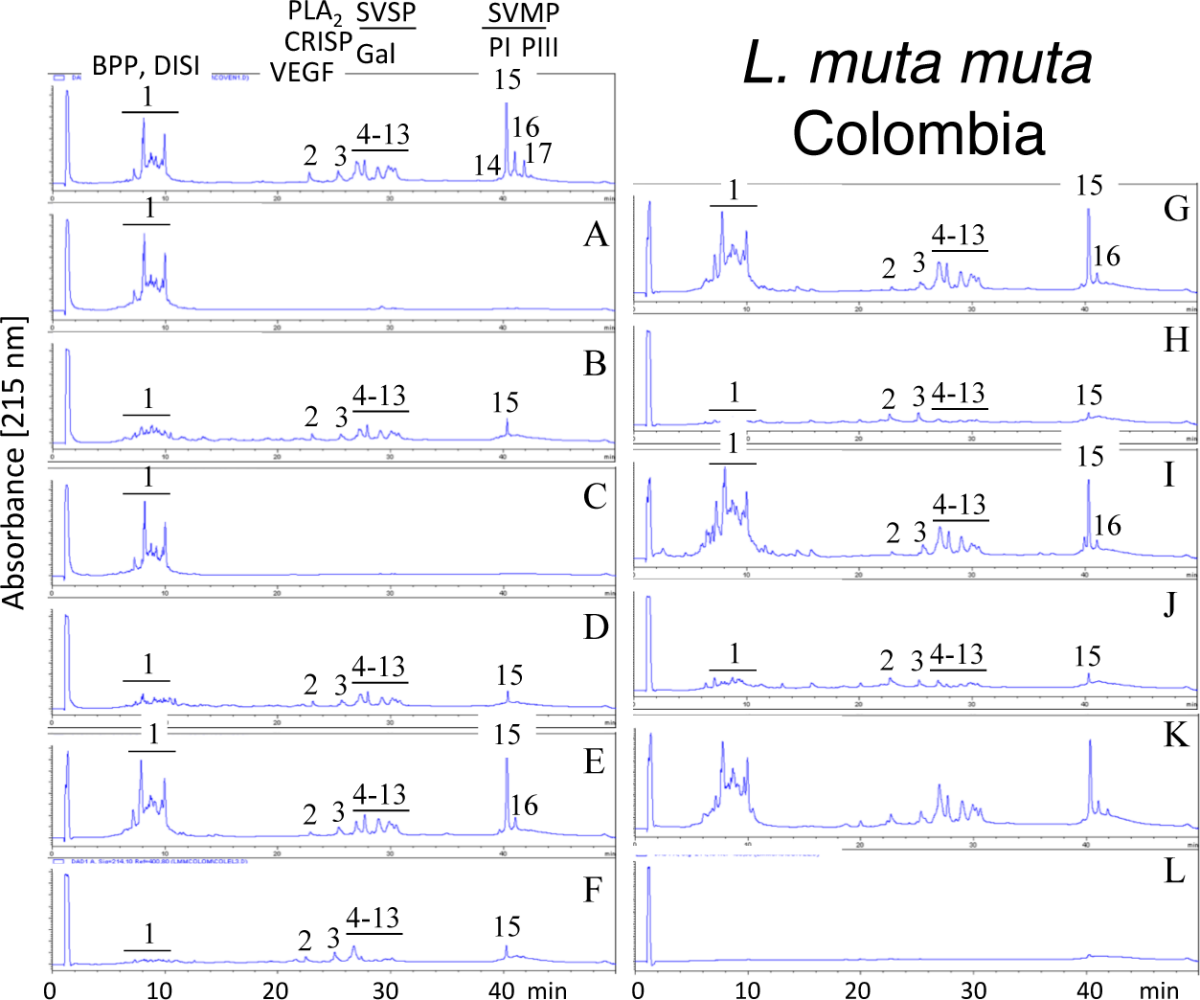
Immunoaffinity chromatography-based antivenomic analysis of the immunoreactivity of polyspecific and monospecific antivenoms towards the venom of *L*. *muta muta* from Colombia. Panels display reverse-phase separations of whole venom components, and non-retained (**A**, **C**, **E**, **G**, and **I**) and the retained (**B**, **D**, **F**, **H**, and **J**) fractions recovered, respectively, from the affinity columns of immobilized BCL, BL, AL, AB, and AC antivenoms. Panels **K** and **L**, non-retained and retained venom fractions by immobilized equine control immunoglobulins. CRISP, cysteine-rich secretory protein. Other protein acronyms as in the legend of Fig 2.

**Fig 5:**
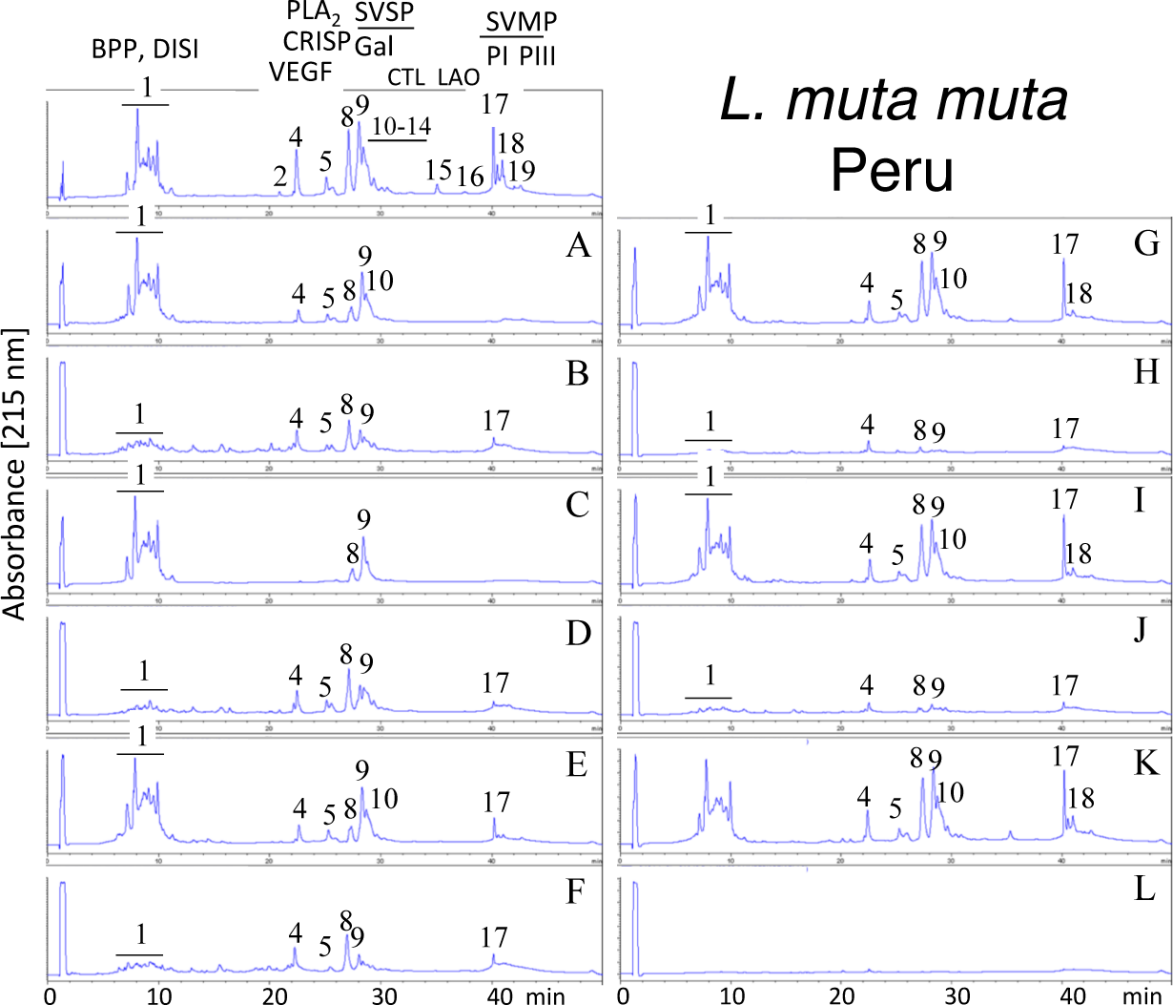
Immunoaffinity chromatography-based antivenomic analysis of the immunoreactivity of polyspecific and monospecific antivenoms towards the venom of *L*. *muta muta* from Peru. Panels display reverse-phase separations of whole venom components, and non-retained (**A**, **C**, **E**, **G**, and **I**) and the retained (**B**, **D**, **F**, **H**, and **J**) fractions recovered, respectively, from the affinity columns of immobilized BCL, BL, AL, AB, and AC antivenoms. Panels **K** and **L**, non-retained and retained venom fractions by immobilized equine control immunoglobulins. CRISP, cysteine-rich secretory protein; CTL, C-type lectin-like; other acronyms as in the legend of Fig 2.

**Fig 6:**
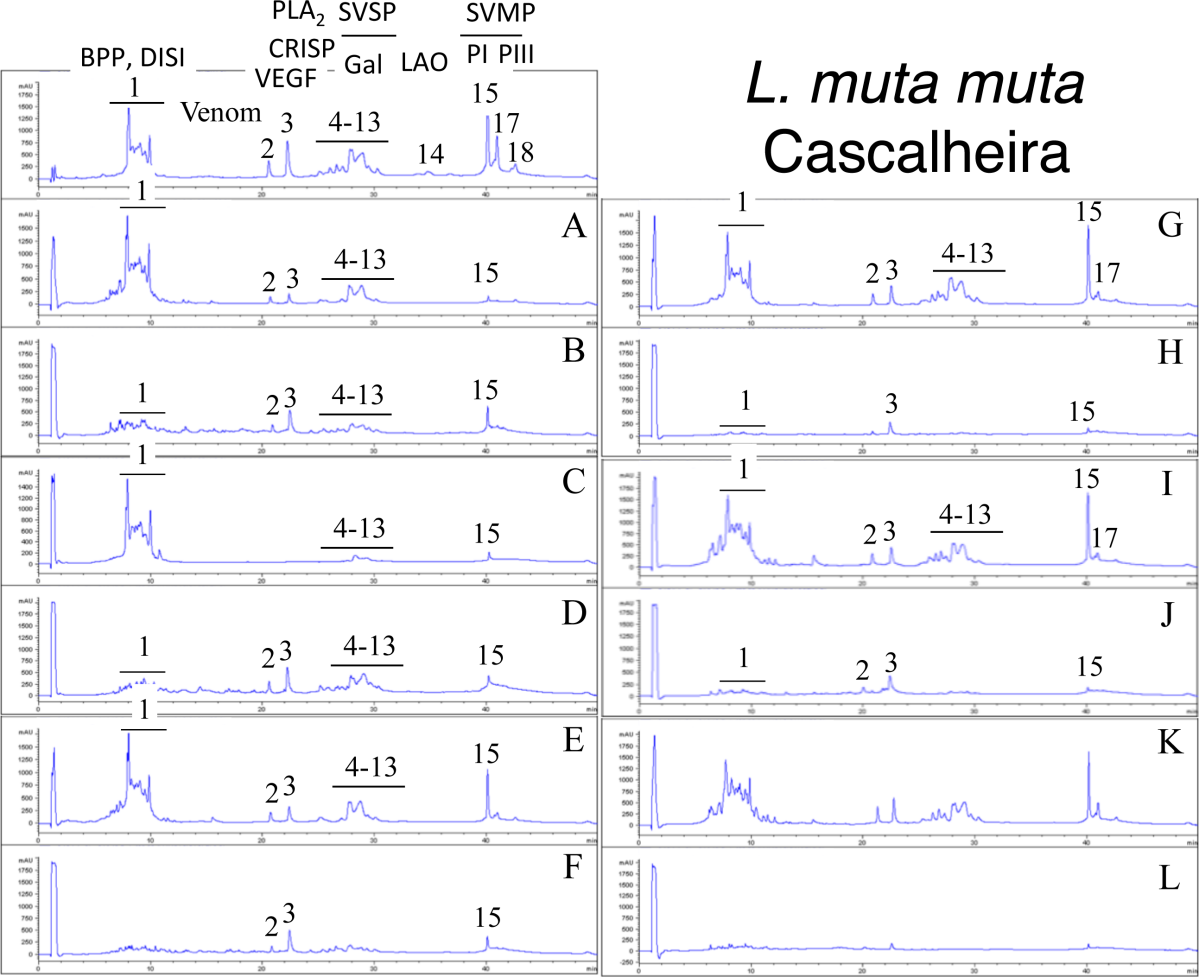
Immunoaffinity chromatography-based antivenomic analysis of the immunoreactivity of polyspecific and monospecific antivenoms towards the venom of *L*. *muta muta* from Cascalheira (Brazil). Panels display reverse-phase separations of whole venom components, and non-retained (**A**, **C**, **E**, **G**, and **I**) and the retained (**B**, **D**, **F**, **H**, and **J**) fractions recovered, respectively, from the affinity columns of immobilized BCL, BL, L, B, and C antivenoms. Panels **K** and **L**, non-retained and retained venom fractions by immobilized equine control immunoglobulins. CRISP, cysteine-rich secretory protein; other acronyms as in the legend of Fig 2.

**Fig 7:**
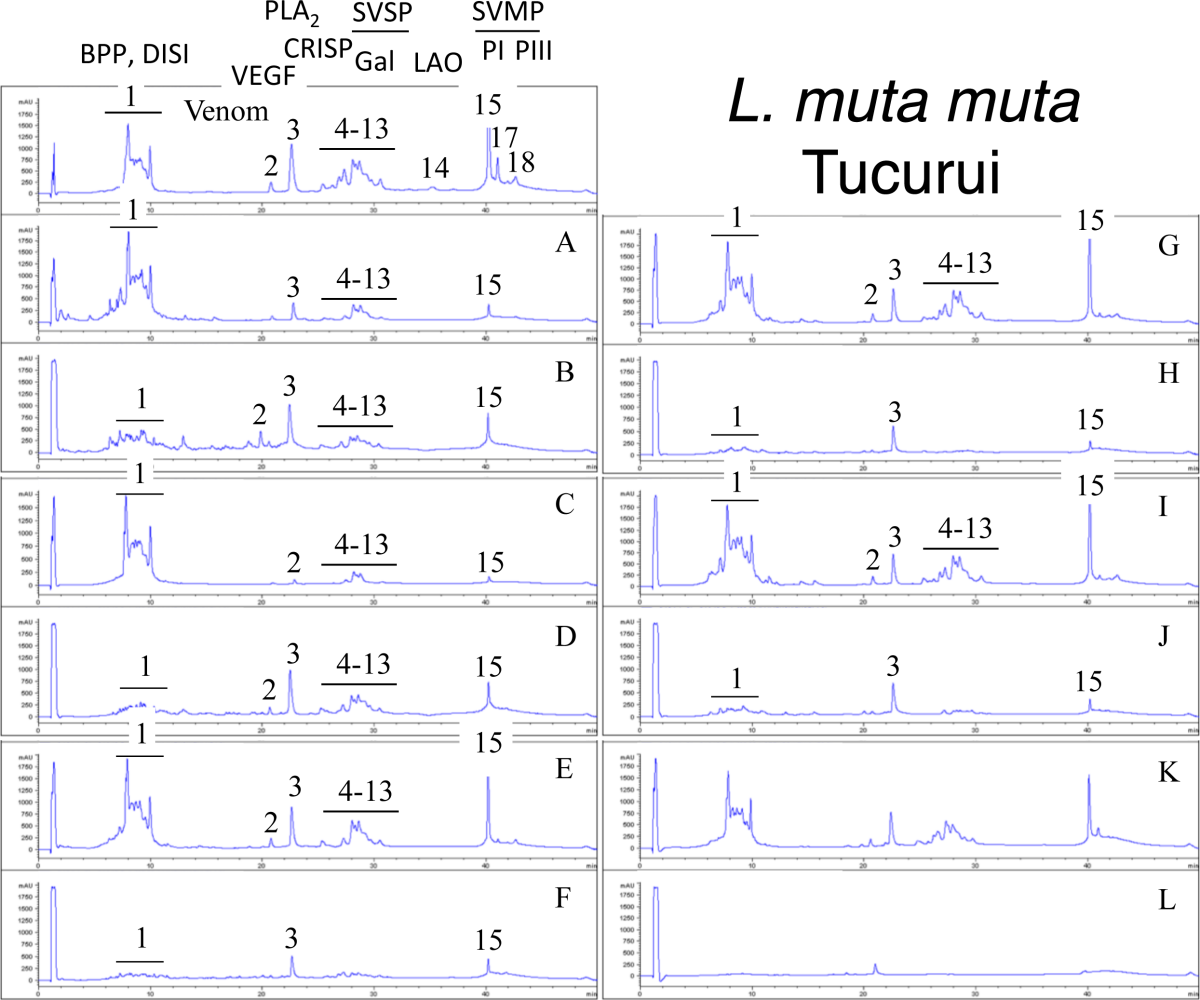
Immunoaffinity chromatography-based antivenomic analysis of the immunoreactivity of polyspecific and monospecific antivenoms towards the venom of *L*. *muta muta* from Tucurui (Brazil). Panels display reverse-phase separations of whole venom components, and non-retained (**A**, **C**, **E**, **G**, and **I**) and the retained (**B**, **D**, **F**, **H**, and **J**) fractions recovered, respectively, from the affinity columns of immobilized BCL, BL, AL, AB, and AC antivenoms. Panels **K** and **L**, non-retained and retained venom fractions by immobilized equine control immunoglobulins. CRISP, cysteine-rich secretory protein; other acronyms as in the legend of Fig 2.

**Fig 8:**
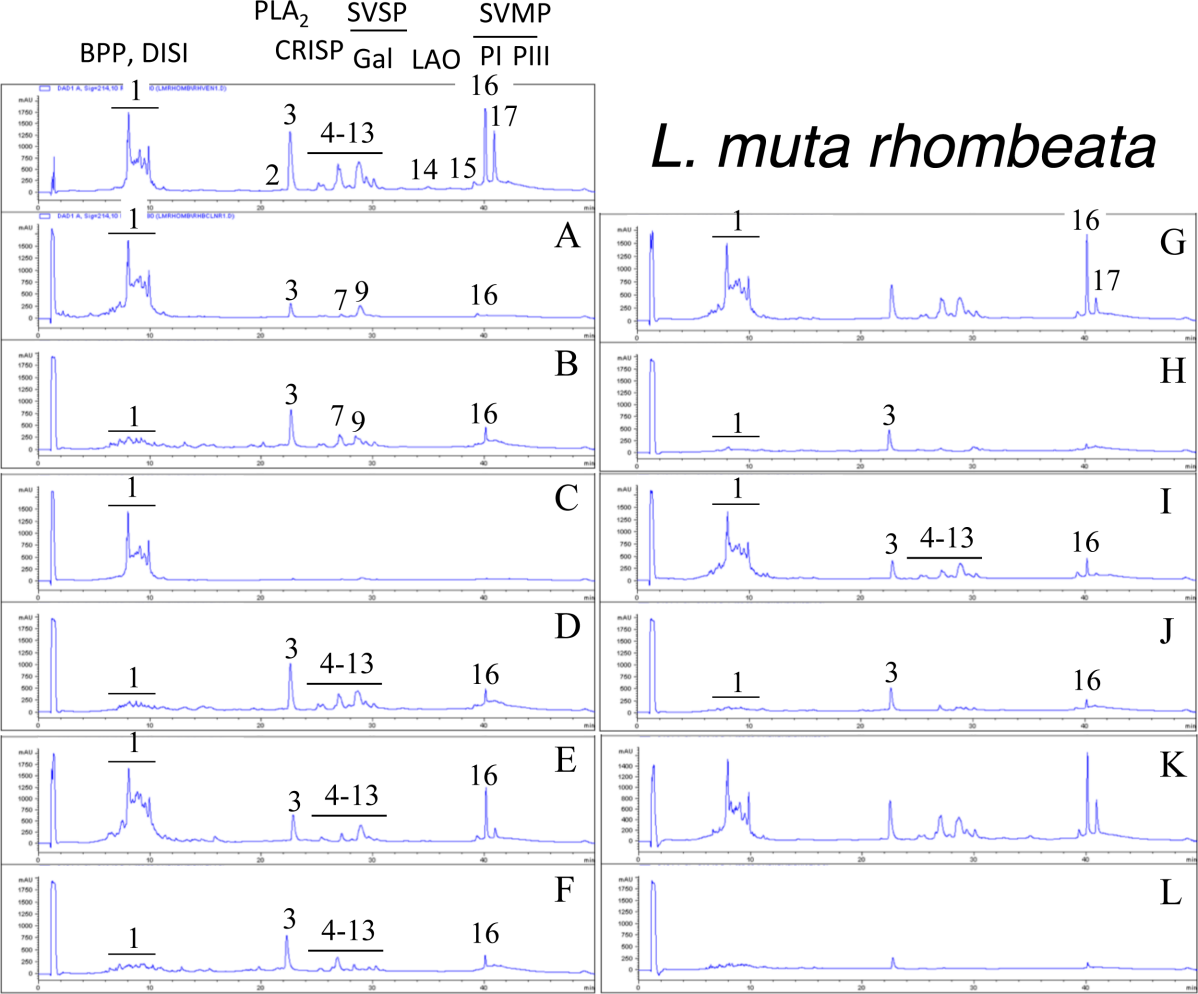
Immunoaffinity chromatography-based antivenomic analysis of the immunoreactivity of polyspecific and monospecific antivenoms towards the venom of *L*. *muta rhombeata* from Brazil. Panels display reverse-phase separations of whole venom components, and non-retained (**A**, **C**, **E**, **G**, and **I**) and the retained (**B**, **D**, **F**, **H**, and **J**) fractions recovered, respectively, from the affinity columns of immobilized BCL, BL, AL, AB, and AC antivenoms. Panels **K** and **L**, non-retained and retained venom fractions by immobilized equine control immunoglobulins. CRISP, cysteine-rich secretory protein; other acronyms as in the legend of Fig 2.

Second generation antivenomics [36] was applied to complement the ELISA and Western blot analyses of the immunoreactivity of the lachesic antivenoms towards the panel of *Lachesis* venoms used for this study. Figs 2–8 display the immunoaffinity chromatography-based antivenomic profiles of commercial BCL and BL polyspecific antivenoms and monospecific B, L, and C experimental antivenoms towards the venoms of *L*. *stenophrys* and *L*. *melanocephala* from Costa Rica, *L*. *muta muta* from Colombia, Peru, and the Brazil regions of Cascalheria, Tucurui, and *L*. *muta rhombeata* from Recife, Brazil. The results show impaired immunocapturing ability of the early eluting chromatographic fractions comprising bradykinin-potentiating-like peptides (BPP-like) by all the antivenoms. Although together these fractions account for about 1/3 by weight of total venom components, previous investigations have shown that the intraperitoneal administration of an amount of BPP-like peptides contained in 10–24 LD_50_s of venom induced neither a significant change in the mean arterial blood pressure of mice, nor signs of abnormal behavior, or histopathological alterations in heart and lungs [25]. These observations strongly suggest that, despite being a major venom component, the BPP-like peptides by themselves may not represent a serious clinical concern in the treatment of *Lachesis* envenomings. The interpretation of these results has to consider that certain antigens may become denatured during reverse-phase separation and, henceforth, some conformational epitopes might be lost.

Except for the BPPs, both polyspecific antivenoms efficiently immunocaptured all the components from *L*. *stenophrys* (Fig 2), *L*. *m*. *muta* (Colombia) (Fig 4) and *L*. *m*. *rhombeata* (Fig 8) venoms. In addition, the BL antivenom immunocaptured the venom components of *L*. *m*. *muta* from the Brazilian localities Cascalheira (Fig 6) and Tucurui (Fig 7). The apparent low recovery of PI- and PIII-SVMPs (eluting from the RP-HPLC column at 40–42 min) in the immunoaffinity captured fractions of the BCL and BL affinity columns (Figs 2–8, panels B and D, respectively) may be ascribed to the high affinity of these venom proteins for the antivenom molecules, as has been demonstrated in a previous work [25].

The worst immunocapturing profile of BCL and BL antivenoms was obtained using *L*. *m*. *muta* from Peru (Fig 5), where Gal-lectin [Q9PSM4] eluting in peak 9 (Fig 5) was essentially (>85%) found in the non-binding fraction. The BCL antivenom also showed limited binding capability towards Gal-lectin [Q9PSM4] and serine proteinase [P33589] from *L*. *m*. *muta* from Cascalheira (peaks 9 and 11, respectively, Fig 6A; 65% of each proteins found in the not retained fraction) and Tucurui (peaks 8 and 10, respectively, Fig 7A, 53% not immunocaptured), and the PLA_2_ molecule eluting in peak 3 of *L*. *m*. *muta* from Tucurui (Fig 7A). 27% of this protein was not immunocaptured by the BCL antivenom.

Monospecific antivenoms showed significantly more limited immunorecognition profiles than BCL and BL antivenoms toward venoms of all *Lachesis* taxa investigated. The three monospecific antivenoms, but particularly the anti-crotalic (AC) antivenom, exhibited poor binding ability towards most venom proteins, including PLA_2_s, CRISP, Gal-lectin, SVSPs, PI- and PIII-SVMPs and LAO. The average toxin immunocapturing activity of this monospecific antivenom was 16% (*L*. *stenophrys*), 21% (*L*. *melanocephala*,), 21% (*L*. *m*. *muta* Colombia), 9% (*L*. *m*. *muta* Peru), 9% (*L*. *m*. *muta* Cascalheira), 17% (*L*. *m*. *muta* Tucurui), and 19% (*L*. *m*. *rhombeata*) (panels I of Figs 2–8, respectively).

Although a comparison of the levels of immune recognition gathered from antivenomics with the *in vivo* neutralization capacity of an antivenom is not straightforward, since both experiments involve radically different protocols, in our experience, an immunocapturing capability of ≥25% of total viperid venom proteins correlates with a good outcome in *in vivo* neutralization tests [48–51]. As a whole, the antivenomics evidence reinforce our view that both polyspecific BCL and BL antivenoms are likely to be effective in the neutralization of heterologous congeneric venoms, thus supporting their use for the treatment of *Lachesis* envenomings throughout the range of distribution of these snakes. In addition, the fact that antivenoms BCL, and particularly BL, are more effective than the monospecific AL antivenom, even against the homologous *L*. *stenophrys* venom, seems to indicate that the inclusion of botropic venoms in the immunization mixture aided in the generation of antibodies exhibiting paraspecificity against *Lachesis* toxins. This combination of immunogens seems to be a more appropriate formulation than a single venom for the treatment of envenomings by *Lachesis* species.

### Neutralization of enzymatic and toxic activities of *Lachesis* venoms

Standard neutralization assays were performed to assess the extent of neutralization of proteolytic, hemorrhagic, procoagulant and lethal activities [39–41]. Despite the fact that all antivenoms were standardized for having a protein concentration of 50 mg/mL, two aspects need to be considered when comparing the values of neutralization experiments: (a) Most antivenoms are made of whole IgG molecules, whereas one of them is made of F(ab’)_2_ fragments; hence, for the same amount of protein, the number of molecules present in an IgG antivenom is 1.5 times lower than in a F(ab`)_2_ antivenom; and (b) only an unknown proportion of all IgGs or F(ab’)_2_ fragments are specific against venom components. Hence, quantitative conclusions drawn by comparing the neutralizing abilities of different type of antivenoms should be regarded as gross estimates.

### Proteolytic activity

The BCL and the BL therapeutic antivenoms, and the monospecific AL antivenom effectively neutralized the proteolytic activity of venoms from the 7 *Lachesis* taxa investigated (Table 2). The BL antivenom showed higher neutralization activity than the other antivenoms used in this study (Table 2). The AC monospecific antivenom was only able to neutralize the proteolytic activity of *L*. *melanocephala* venom (Table 2). The AB monospecific antivenom was unable to neutralize the proteolytic activity of any of the venoms (Table 2).

**Table 2:** Neutralization of the proteolytic activity of *Lachesis* venoms by polyspecific BCL and BL and monospecific antivenoms.

		ED_50_ (μL/mg)			
Venom	BCL	BL	AL	AB	AC
***L*. *stenophrys***	876.1 ± 21.32 ^a^*	473.0 ± 9.19 ^b^	2183.2 ± 13.72^c^	nn^1^	nn^1^
***L*. *melanocephala***	229.0 ± 3.67 ^a^	169.9 ± 4.73 ^b^	651.2 ± 34.72 ^c^	nn^2^	3334.9 ± 377.06^d^
***L*. *m*. *muta Colombia***	789.1 ± 21.67 ^a^	293.5 ± 8.04 ^b^	1736.5 ± 36.75 ^c^	nn^1^	nn^1^
***L*. *m*. *muta Perú***	375.9 ± 12.47 ^a^	148.5 ± 8.99 ^b^	1240.1 ± 49.19 ^c^	nn^1^	nn^1^
***L*. *m*. *muta Cascalheira***	538.3 ± 26.50 ^a^	241.5 ± 11.52 ^b^	1616.1 ± 79.33 ^c^	nn^1^	nn^1^
***L*. *m*. *muta Tucurui***	861.9 ± 35.00 ^a^	221.9 ± 6.70 ^b^	1375.6 ± 18.30 ^c^	nn^1^	nn^1^
***L*. *m*. *rhombeata Recife***	592.3 ± 19.98 ^a^	219.2 ± 5.36 ^b^	1422.2 ± 152.25 ^c^	nn^1^	nn^1^

^1^ nn, no neutralization at ratio 4000 μL antivenom/mg venom.

^2^ No neutralization at ratio 3600 μL antivenom/mg venom.

BCL: polyspecific anti-bothropic, anti-crotalic, anti-lachesic ICP antivenom; BL: anti-bothropic and anti-lachesic antivenom from Instituto Vital Brazil; AL, monospecific anti-lachesic antivenom; AB, monospecific anti-bothropic antivenom; AC, monospecific anti-crotalic antivenom.

*Values with different superscripts are significantly different for the various antivenoms against a single venom (p<0.05).

### Hemorrhagic activity

The BCL and BL antivenoms, and the monospecific L antivenom effectively neutralized the hemorrhagic activity of all the *Lachesis* venoms studied (Table 3). The BL antivenom showed the highest neutralization capacity of the hemorrhagic activity than any of the other antivenoms used in this study (Table 3). The monospecific AB antivenom was only able to neutralize the hemorrhagic activity of *L*. *stenophrys* and *L*. *melanocephala* venoms, whereas the monospecific AC antivenom was unable to neutralize the hemorrhagic activity of any of the venoms (Table 3).

**Table 3:** Neutralization of the hemorrhagic effect of *Lachesis* venoms by polyspecific BCL and BL and monospecific antivenoms.

	ED_50_ (μL/mg)				
Venom/Antivenom	BCL	BL	AL	AB	AC
***L*. *stenophrys***	238.7 ± 25.57 ^a^*	84.7 ± 3.23^b^	508.9 ± 63.91^c^	2289.0 ± 253.90^d^	nn^1^
***L*. *melanocephala***	371.3 ± 5.80 ^a^	120.8 ± 10.3 ^b^	591.3 ± 9.70 ^c^	3258.3 ± 210.40 ^d^	nn^1^
***L*. *m*. *muta Colombia***	224.6 ± 26.48 ^a^	88.3 ± 11.72 ^b^	777.9 ± 105.43 ^c^	nn^1^	nn^1^
***L*. *m*. *muta Perú***	251.7 ± 26.23 ^a^	43.9 ± 11.00 ^b^	917.1 ± 245.13 ^c^	nn^1^	nn^1^
***L*. *m*. *muta Cascalheira***	291.3 ± 30.10 ^a^	68.6 ± 0.50 ^b^	2564.8 ± 239.40 ^c^	nn^1^	nn^1^
***L*. *m*. *muta Tucurui***	225.2 ± 53.18 ^a^	111.9 ± 18.46 ^b^	472.4 ± 53.08 ^c^	nn^1^	nn^1^
***L*. *m*. *rhombeata Recife***	314.5 ± 7.52 ^a^	53.2 ± 9.90 ^b^	581.5 ± 41.54 ^c^	nn^1^	nn^1^

^1^ nn, no neutralization at ratio 4000 μL antivenom/mg venom.

BCL: polyspecific anti-bothropic, anti-crotalic, anti-lachesic ICP antivenom; BL: anti-bothropic and anti-lachesic antivenom from Instituto Vital Brazil; AL, monospecific anti-lachesic antivenom; AB, monospecific anti-bothropic antivenom; AC, monospecific anti-crotalic antivenom.

*Values with different superscripts are significantly different for the various antivenoms against a single venom (p<0.05).

### Coagulant activity

The BCL and BL polyspecific antivenoms, and the monospecific L antivenom effectively neutralized the coagulant activity of *Lachesis* venoms from the seven bushmaster taxa sampled (Table 4). The BL antivenom showed the highest coagulant neutralization activity than any of the other antivenoms use in this study (Table 4). On the other hand, neither the AB nor the AC monospecific antivenoms were able to neutralize the coagulant activity of any of the *Lachesis* venoms used in this study (Table 4). These data agree with a previous work showing the inefficacy of monospecific bothropic antivenom in the neutralization of the coagulation activity of *L*. *m*. *muta* venom [54].

**Table 4:** Neutralization of coagulant effect of *Lachesis* venoms by polyspecific BCL and BL and monospecific antivenoms.

	ED (μL/mg)				
Venom/Antivenom	BCL	BL	AL	AB	AC
***L*. *stenophrys***	708.1 ± 10.34^a^*	141.1 ± 4.73 ^b^	651.6 ± 14.34 ^c^	nn^1^	nn^1^
***L*. *melanocephala***	514.2 ± 2.08 ^a^	109.9 ± 4.04 ^b^	660.9 ± 3.13 ^c^	nn^1^	nn^1^
***L*. *m*. *muta Colombia***	752.2 ± 36.7 ^a^	336.0 ± 21.3 ^b^	1039.6 ± 15.8 ^c^	nn^1^	nn^1^
***L*. *m*. *muta Perú***	1322.7 ± 30.1 ^a^	209.8 ± 10.2 ^b^	1701.1 ± 52.9 ^c^	nn^1^	nn^1^
***L*. *m*. *muta Cascalheira***	1341.3 ± 175.4 ^a^	222.8 ± 8.80 ^b^	1269.1 ± 78.40 ^a^	nn^1^	nn^1^
***L*. *m*. *muta Tucurui***	726.5 ± 24.86 ^a^	138.4 ± 1.11 ^b^	836.4 ± 3.72 ^c^	nn^1^	nn^1^
***L*. *m*. *rhombeata Recife***	846.2 ± 6.60 ^a^	463.8 ± 1.50 ^b^	1563.1± 32.3 ^c^	nn^1^	nn^1^

^1^ nn, no neutralization at ratio 4000 μL antivenom/mg venom.

BCL: polyspecific anti-bothropic, anti-crotalic, anti-lachesic ICP antivenom; BL: anti-bothropic and anti-lachesic antivenom from Instituto Vital Brazil; AL, monospecific anti-lachesic antivenom; AB, monospecific anti-bothropic antivenom; AC, monospecific anti-crotalic antivenom.

*Values with different superscripts are significantly different for various antivenoms against a single venom (p<0.05).

### Lethal activity

At the antivenom/venom ratio of 500 μL antivenom/mg venom, the BCL and BL polyspecific antivenoms and the monospecific L antivenom, effectively neutralized the lethal activity of the seven *Lachesis* venoms investigated (Table 5). The monospecific AB antivenom only neutralized the lethal activity of *L*. *stenophrys*, *L*. *muta muta* (Cascalheira) and *L*. *muta rhombeata* (Recife) (Table 5), while the monospecific AC antivenom was unable to neutralize the lethal activity of any of the *Lachesis* venoms studied at the ratio of 500 μL antivenom/mg venom (Table 5).

**Table 5:** Neutralization of lethality of *Lachesis* venoms by polyspecific BCL and BL and monospecific antivenoms*.

Venom/Antivenom	BCL	BL	AL	AB	AC
***L*. *stenophrys***	*+*	*+*	*+*	*+*	*-*
***L*. *melanocephala***	*+*	*+*	*+*	*-*	*-*
***L*. *m*. *muta Colombia***	*+*	*+*	*+*	*-*	*-*
***L*. *m*. *muta Peru***	*+*	*+*	*+*	*-*	*-*
***L*. *m*. *muta Cascalheira***	*+*	*+*	*+*	*+*	*-*
***L*. *m*. *muta Tucurui***	*+*	*+*	*+*	*-*	*-*
***L*. *m*. *rhombeata Recife***	*+*	*+*	*+*	*+*	*-*

*Results are represented by capacity of antivenoms to protect mice at a ratio of 500 μL antivenom/mg venom.

BCL: polyspecific anti-bothropic, anti-crotalic, anti-lachesic ICP antivenom; BL: anti-bothropic and anti-lachesic antivenom from Instituto Vital Brazil; AL, monospecific anti-lachesic antivenom; AB, monospecific anti-bothropic antivenom; AC, monospecific anti-crotalic antivenom.

(+) indicates neutralization and (-) indicates lack of neutralization.

### Concluding remarks

Snakes from the *Lachesis* genus cause severe envenomings in humans and are widely distributed in a variety of habitats ranging from the Caribbean coast of Central America to the Atlantic rainforest of Brazil. Based on the high conservation of the overall protein composition of *Lachesis* venoms and their qualitatively similar pathophysiological profile observed in experimental envenomings and clinical settings we have suggested that antivenoms generated against any conspecific *Lachesis* venom may exhibit paraspecific protection against the toxic activities of all other *Lachesis* species. Combining immunochemical methods, second generation antivenomics, and venom neutralization tests we have unveiled the efficacy of two therapeutic polyvalent antivenoms and three experimental monospecific antivenoms to recognize the complete proteomes and neutralize the hemorrhagic, coagulant, proteolytic and lethal activities from three different *Lachesis* species from different geographic populations. The results demonstrate that antivenoms raised by immunizing horses with the venoms of different *Lachesis* species are effective in the neutralization of congeneric venoms not used in the immunization mixture, indicating that they could be used equivalently for the clinical treatment of any lachesic envenoming. Owing to the similar clinical presentations of envenomings by *Lachesis* sp. and *Bothrops* sp., the use of polyvalent antivenoms which include the *Lachesis* component is therefore recommended in Latin America.

## Supporting information

S1 Fig Titration curves for mono and polyspecific antivenoms against *Lachesis* venoms. Antivenoms were serially diluted by a factor of 3 (starting from a dilution of 1/500) and tested by ELISA against the following crude *Lachesis* venoms: *L*. *stenophrys* from Costa Rica (**A**), *L*. *melanocephala* from Costa Rica (**B**), *L*. *muta muta* from Colombia (**C**), Peru (**D**), the Brazil regions of Cascalheria (**E**), Tucurui (**F**), and *L*. *muta rhombeata* from Recife, Brazil (**G**). Antivenom acronyms, BCL, polyspecific anti-bothropic, anti-crotalic, anti-lachesic antivenom from Instituto Clodomiro Picado (Cr); BL, anti-bothropic and anti-lachesic antivenom from Instituto Vital Brazil, Niterói, Brazil; AL, monoespecific anti-lachesic antivenom; AB, monoespecific anti-bothropic antivenom; AC, monoespecific anti-crotalic antivenom. Each point represents the mean ± SD of three independent determinations.
(DOCX)

S1 Table Proteomic identification of 2DE resolved proteins from Costa Rican *L*. *stenophrys* venom. (DOCX)

S2 Table 2DE-separated *L*. *stenophrys* venom protein spots recognized by mono and polyspecific antivenoms. (DOCX)
