# Supplementary material for: Cross-reactivity, antivenomics, and neutralization of toxic activities of Lachesis venoms by polyspecific and monospecific antivenoms
Source: PLoS Negl Trop Dis. 2017 Aug 7;11(8):e0005793. doi: 10.1371/journal.pntd.0005793 (PMC5560760; doi:10.1371/journal.pntd.0005793)
Supplement: S1 Fig — Antivenoms were serially diluted by a factor of 3 (starting from a dilution of 1/500) and tested by ELISA against the following crude Lachesis venoms: L. stenophrys from Costa Rica (A), L. melanocephala from Costa Rica (B), L. muta muta from Colombia (C), Peru (D), the Brazil regions of Cascalheria (E), Tucurui (F), and L. muta rhombeata from Recife, Brazil (G). Antivenom acronyms, BCL, polyspecific anti-bothropic, anti-crotalic, anti-lachesic antivenom from Instituto Clodomiro Picado (Cr); BL, anti-bothropic and anti-lachesic antivenom from Instituto Vital Brazil, Niterói, Brazil; AL, monoespecific anti-lachesic antivenom; AB, monoespecific anti-bothropic antivenom; AC, monoespecific anti-crotalic antivenom. Each point represents the mean ± SD of three independent determinations. (DOCX) [file pntd.0005793.s001.docx]

**S1 Figure**


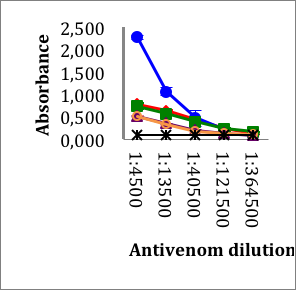


A


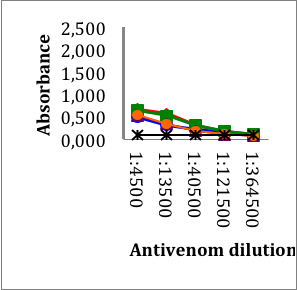


B


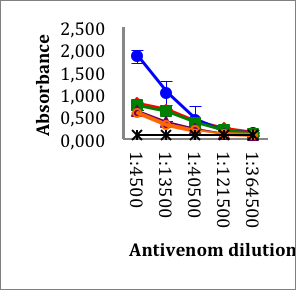


C


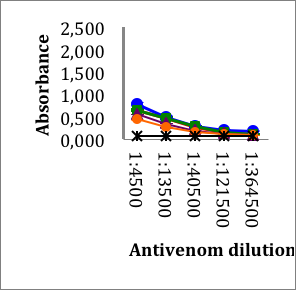


D


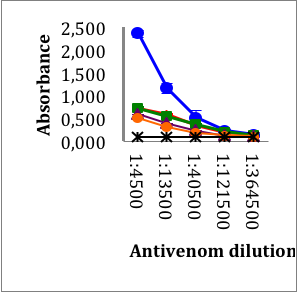


E


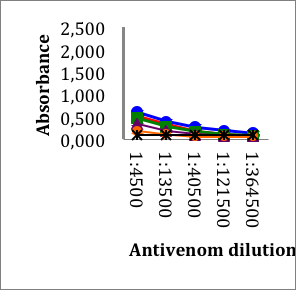


F


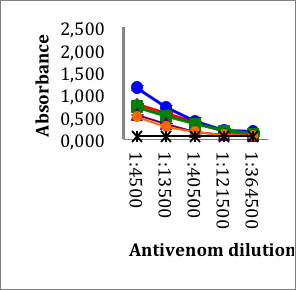


G

Titration curves for mono and polyspecific antivenoms against *Lachesis* venoms. Antivenoms were serially diluted by a factor of 3 (starting from a dilution of 1/500) and tested by ELISA against the following crude *Lachesis* venoms: *L. stenophrys* from Costa Rica (**A**), *L. melanocephala* from Costa Rica (**B**), *L. muta muta* from Colombia (**C**), Peru (**D**), the Brazil regions of Cascalheria (**E**), Tucurui (**F**), and *L. muta rhombeata* from Recife, Brazil (**G**). Antivenom acronyms, BCL, polyspecific anti-bothropic, anti-crotalic, anti-lachesic antivenom from Instituto Clodomiro Picado (Cr); BL, anti-bothropic and anti-lachesic antivenom from Instituto Vital Brazil, Niterói, Brazil; AL, monoespecific anti-lachesic antivenom; AB, monoespecific anti-bothropic antivenom; AC, monoespecific anti-crotalic antivenom. Each point represents the mean ± SD of three independent determinations.
