# Supplementary material for: Cross-reactivity, antivenomics, and neutralization of toxic activities of Lachesis venoms by polyspecific and monospecific antivenoms
Source: PLoS Negl Trop Dis. 2017 Aug 7;11(8):e0005793. doi: 10.1371/journal.pntd.0005793 (PMC5560760; doi:10.1371/journal.pntd.0005793)
Supplement: S1 Table — (DOCX) [file pntd.0005793.s002.docx]

**S1 Table.** Proteomic identification of 2DE resolved proteins from Costa Rican *L. stenophrys* venom

| **Spot** | **Rel. mol. mass** | **% proteins**  **in gel** | **IP** | **m/z** | **z** | **N** | **Protein family** |
| --- | --- | --- | --- | --- | --- | --- | --- |
| **1** |  | 0.233 |  | - | - | - | nd |
| **2** | 70.9-99.0 | 0.010 | 6.5 | 1037.4 | 1 | FWEDDGIR | LAO (J7H670) |
| **3** | 70.9-99.0 | 0.828 | 6.4 | 1486.6 | 1 | ETDYEEFLEIAK | LAO (J7H670) |
|  |  |  |  | 1037.4 | 1 | FWEDDGIR | LAO (J7H670) |
| **4** | 70.9-99.0 | 1.547 | 6.3 | 1037.5 | 1 | FWEDDGIR | LAO (J7H670) |
|  |  |  |  | 1486.7 | 1 | ETDYEEFLEIAK | LAO (J7H670) |
| **5** |  | 0.469 |  | - | - | - | nd |
| **6** |  | 0.081 |  | - | - | - | nd |
| **7** |  | 0.098 |  | - | - | - | nd |
| **8** |  | 0.069 |  | - | - | - | nd |
| **9** |  | 0.298 |  | - | - | - | nd |
| **10** | 57.2-69.2 | 0.463 | 6.1 | 1957 | 1 | YLISHTPQCILNEPLR | SVMP-III (C5H5D4) |
|  |  |  |  | 2888.4 | 1 | DIVSPPICGNELLELEEECDCGSPR | SVMP-III (AEJ31193) |
| **11** | 57.2-69.2 | 0.203 | 6 | 2888.4 | 1 | DIVSPPICGNELLELEEECDCGSPR | SVMP-III (AEJ31193) |
| **12** | 61.4-74.4 | 1.083 | 5.9 | 2239.9 | 1 | QHSWVECESGECCEQCK | SVMP-III (ABG26980) |
| **13** | 61.4-74.4 | 0.628 | 5.8 | 1749.8 | 1 | ELLEVGEECDCGSPR | SVMP-III (Q92043) |
|  |  |  |  | 1299.7 | 1 | YVELVVVADHR | SVMP-III (Q7SZD9) |
|  |  |  |  | 2888.3 | 1 | DIVSPPICGNELLELEEECDCGSPR | SVMP-III (AEJ31993) |
| **14** | 69.2 | 0.128 | 5.7 | 1957 | 1 | YLISHTPQCILNEPLR | SVMP-III (C5H5D4) |
| **15** | 64.4 | 0.097 | 5.7 | 1957 | 1 | YLISHTPQCILNEPLR | SVMP-III (C5H5D4) |
| **16** |  | 0.226 |  | - | - | - | nd |
| **17** | 71.7 | 0.161 | 5.3 | 1340.7 | 1 | TPQCILNEPLR | SVMP-III (C5H5D4) |
| **18** |  | 0.079 |  | - | - | - | nd |
| **19** |  | 0.754 |  | - | - | - | nd |
| **20** |  | 0.942 |  | - | - | - | nd |
| **21** |  | 0.332 |  | - | - | - | nd |
| **22** | 42.0-53.2 | 4.220 | 6.6 | 1603.9 | 1 | VHEIVNTINVFYR | SVMP-III (C9E1R9) |
| **23** | 45.6-53.2 | 1.337 | 6.5 | 1733.7 | 1 | VLCAGVLEGGIDTCNR | SVSP (B0VXU2) |
| **24** |  | 0.437 |  | - | - | - | nd |
| **25** | 46.7-57.2 | 2.090 | 6.3 | 1512.7 | 1 | VIGGDECNINEHR | SVSP (P33589) |
|  |  |  |  | 1733.9 | 1 | VLCAGVLEGGIDTCNR | SVSP (P33589) |
| **26** |  | 0.068 |  | - | - | - | nd |
| **27** | 46.7-53.2 | 1.588 | 6.1 | 1512.7 | 1 | VIGGDECNINEHR | SVSP (P33589) |
|  |  |  |  | 1733.9 | 1 | VLCAGVLEGGIDTCNR | SVSP (P33589) |
|  |  |  |  | 1190.6 | 1 | WDEDIMLIR | SVSP (P33589) |
| **28** |  | 0.063 |  | - | - | - | nd |
| **29** | 47.8-54.5 | 1.534 | 5.9 | 1733.8 | 1 | VLCAGVLEGGIDTCNR | SVSP (P33589) |
|  |  |  |  | 1512.7 | 1 | VIGGDECNINEHR | SVSP (P33589) |
| **30** |  | 0.021 |  | - | - | - | nd |
| **31** | 47.8-54.5 | 1.174 | 5.7 | 1733.8 | 1 | VLCAGVLEGGIDTCNR | SVSP (B0VXU2) |
| **32** |  | 0.476 |  | - | - | - | nd |
| **33** |  | 0.135 |  | - | - | - | nd |
| **34** |  | 0.822 |  | - | - | - | nd |
| **35** |  | 0.149 |  | - | - | - | nd |
| **36** |  | 0.267 |  | - | - | - | nd |
| **37** |  | 0.118 |  | - | - | - | nd |
| **38** |  | 0.042 |  | - | - | - | nd |
| **39** | 34.7 | 0.529 | 7.1 | 1581.7 | 1 | CANINILDYAVCR | SVSP (T1DE97) |
|  |  |  |  | 1564.7 | 1 | CANINILDYAVCR | SVSP (T1DE97) |
|  |  |  |  | 1347.6 | 1 | AAYPWWPVTTR | SVSP (T1DE97) |
|  |  |  |  | 1488.7 | 1 | VVGGHPCNINEHR | SVSP (T1E3B5) |
| **40** |  | 1.124 |  | - | - | - | nd |
| **41** | 32.3-41.0 | 1.061 | 6.6 | 1607.5 | 1 | VHEIVNFINGFYR | SVMP-III (Q8JJ50) |
| **42** | 35.5 | 0.667 | 6.5 | 2512.2 | 1 | EIYPDVPHCADINILDHAVCR | SVSP (Q27J47) |
| **43** |  | 0.697 |  | - | - | - | nd |
| **44** |  | 0.763 |  | - | - | - | nd |
| **45** |  | 0.255 |  | - | - | - | nd |
| **46** | 37.2-44.0 | 1.364 | 6 | 1294.7 | 1 | INLLDYEVCR | SVSP (B0VXU2) |
|  |  |  |  | 1622.8 | 1 | CANINLLDYEVCR | SVSP (B0VXU2) |
|  |  |  |  | 1639.9 | 1 | CANINLLDYEVCR | SVSP (B0VXU2) |
|  |  |  |  | 1421.8 | 1 | APYPEFGLPATSR | SVSP (Q91053) |
| **47** | 35.5 | 0.923 | 6 | 1365.7 | 1 | AYPEFGLPATSR | SVSP (T1E6T7) |
|  |  |  |  | 1546.7 | 1 | VFGGDECNINEHR | SVSP (Q27J47) |
|  |  |  |  | 1429.7 | 1 | SLPSSPPSVGSVCR | SVSP (Q27J47) |
|  |  |  |  | 1087.5 | 1 | VPNEDEQTR | SVSP |
|  |  |  |  | 1512.7 | 1 | VIGGDECNINEHR | SVSP (Q27J47) |
| **48** | 40.0 | 1.570 | 5.8 | 1421.8 | 1 | APYPEFGLPATSR | SVSP (Q91053) |
|  |  |  |  | 1512.7 | 1 | VIGGDECNINEHR | SVSP (Q91053) |
| **49** | 35.1 | 1.974 | 5.8 | 2507.1 | 1 | EIYPDVPHCADINILDHAVCR | SVSP (Q27J47) |
|  |  |  |  | 1546.6 | 1 | VFGGDECNINEHR | SVSP (Q27J47) |
|  |  |  |  | 1277.7 | 1 | LALDIEIATYR | SVSP (Q27J47) |
| **50** | 40.0 | 4.047 | 5.6 | 1546.7 | 1 | VFGGDECNINEHR | SVSP (Q27J47) |
|  |  |  |  | 2507.3 | 1 | EIYPDVPHCADINILDHAVCR | SVSP (Q27J47) |
|  |  |  |  | 1421.8 | 1 | APYPEFGLPATSR | SVSP (Q91053) |
| **51** | 35.1 | 3.272 | 5.6 | 1365.6 |  | AYPEFGLPATSR | SVSP (T1E6T7) |
|  |  |  |  | 1512.6 |  | VIGGDECNINEHR | SVSP (T1DP95) |
| **52** |  | 0.091 |  | - | - | - | nd |
| **53** | 41.0 | 1.940 | 5.4 | 1546.7 | 1 | VFGGDECNINEHR | SVSP (Q27J47) |
|  |  |  |  | 2507.2 | 1 | VTYPDVPHCANINILDHAVCR | SVSP (Q27J47) |
| **54** | 35.9 | 0.842 | 5.4 | 1365.7 | 1 | AYPEFGLPATSR | SVSP (T1E6T7) |
|  |  |  |  | 1512.7 | 1 | VIGGDECNINEHR | SVSP (T1E6T7) |
|  |  |  |  | 1429.7 | 1 | SLPSSPPSVGSVCR | SVSP (T1E6T7) |
| **55** |  | 0.079 |  | - | - | - | nd |
| **56** | 38.1 | 0.379 | 5.3 | 1814.8 | 1 | EICECDRDAAICFR | PLA_2_ (P84651) |
|  |  |  |  | 1298.7 | 1 | HLLQFGDLIDK | PLA_2_ (P84651) |
|  |  |  |  | 950.5 | 1 | YWLFPPK | PLA_2_ (T1DH17) |
|  |  |  |  | 1506.5 | 1 | CCFVHDCCYGK | PLA_2_ (T1DH17) |
| **57** | 33.9 | 1.709 | 5.3 | 1498.6 | 1 | VVGGDECNINEHR | SVSP (T2HS34) |
| **58** | 34.7 | 0.323 | 5.2 | 1498.6 | 1 | VVGGDECNINEHR | SVSP (T2HS34) |
| **59** | 36.4 | 0.308 | 5 | 1379.7 | 1 | AAYPEFGLPATSR | SVSP (T1E6T7) |
|  | 38.1 |  |  | 1526.7 | 1 | IIGGDECNINEHR | SVSP (T1E6T7) |
| **60** |  | 4.220 |  | - | - | - | nd |
| **61** |  | 4.364 |  | - | - | - | nd |
| **62** | 28.0 | 17.996 | 6.2-7.5 | 1737.8 | 1 | YIELVVVADHGMFTK | SVMP-I (Q9YI19) |
|  |  |  |  | 1603.8 | 1 | VHEIVNTINVFYR | SVMP-I (Q9YI19) |
|  |  |  |  | 2050.8 | 1 | LRPGAQCAEGLCCDQCR | SVMP-I (Q92119) |
| **63** |  | 0.098 |  | - | - | - | nd |
| **64** | 28.0 | 2.132 | 5 | 1065.5 | 1 | YNGNLNTIR | SVMP-I (Q9IAB0) |
|  |  |  |  | 1047.5 | 1 | YNGNLNTIR | SVMP-I (Q9IAB0) |
|  |  |  |  | 1783.8 | 1 | YIQLVVVADHGMFMK | SVMP-I (P86802) |
| **65** |  | 0.886 |  | - | - | - | nd |
| **66** |  | 0.092 |  | - | - | - | nd |
| **67** |  | 0.215 |  | - | - | - | nd |
| **68** |  | 0.437 |  | - | - | - | nd |
| **69** | 18.6 | 0.766 | 8 | 2050.8 | 1 | LRPGAQCAEGLCCDQCR | SVMP-I (Q92119) |
| **70** |  | 1.310 |  | - | - | - | nd |
| **71** |  | 0.350 |  | - | - | - | nd |
| **72** | 11.9 | 0.565 | 8 | 1486.6 | 1 | ETDYEEFLEIAK | LAO (J7H670) |
|  |  |  |  | 1449.6 | 1 | ADDRNPLGECFR | LAO (J7H670) |
| **73** |  | 0.286 |  | - | - | - | nd |
| **74** |  | 0.191 |  | - | - | - | nd |
| **75** |  | 0.066 |  | - | - | - | nd |
| **76** |  | 0.115 |  | - | - | - | nd |
| **77** | 16.2 | 0.819 | 6.5 | 1486.7 | 1 | ETDYEEFLEIAK | LAO |
| **78** |  | 0.239 |  | - | - | - | nd |
| **79** | 15.4 | 7.592 | 5.3-6.0 | 1298.6 | 1 | HLLQFGDLIDK | PLA_2_ (P84651) |
|  |  |  |  | 2169.8 | 1 | SGFWYYGFYGCYCGLGGR | PLA_2_ (P84651) |
|  |  |  |  | 1814.7 | 1 | EICECDRDAAICFR | PLA_2_ (P84651) |
|  |  |  |  | 1505.5 | 1 | CCFVHDCCYGK | PLA_2_ (P84651) |
|  |  |  |  | 1241.5 | 1 | DFSWEWTDR | C-type lectin (Q9PSM4) |
|  |  |  |  | 950.5 | 1 | YWLFPPK | PLA_2_ (T1DH17) |
| **80** | 14.5 | 3.057 | 5.4-6.0 | 1288.5 | 1 | SCTDYLTWDK | C-type lectin (Q9PSM4) |
|  |  |  |  | 1401.6 | 1 | GQAEVWIGLWDK | C-type lectin (Q9PSM4) |
|  |  |  |  | 1278.5 | 1 | LWNDQVCESK | C-type lectin (Q9PSM4) |
|  |  |  |  | 1916.8 | 1 | YGESLEIAEYISDYHK | C-type lectin (Q9PSM4) |
|  |  |  |  | 1241.5 | 1 | DFSWEWTDR | C-type lectin (Q9PSM4) |
|  |  |  |  | 1572.7 | 1 | EFCVELVSLTGYR | C-type lectin (Q9PSM4) |
| **81** |  | 0.474 |  | - | - | - | nd |
| **82** | 14.7-18.6 | 4.378 | 5.3 | 1298.8 | 1 | HLLQFGDLIDK | PLA_2_ (P84651) |
|  |  |  |  | 1505.6 | 1 | CCFVHDCCYGK | PLA_2_ (P84651) |
|  |  |  |  | 2170 | 1 | SGFWYYGFYGCYCGLGGR | PLA_2_ (P84651) |
|  |  |  |  | 1241.6 | 1 | DFSWEWTDR | Gal-binding lectin (T2HP42) |
|  |  |  |  | 950.6 | 1 | YWLFPPK | PLA_2_ (T1DH17) |
| **83** | 15.4 | 0.548 | 5.1 | 2619.2 | 1 | KDIYTYSEENGAIVCGGDNPCKK | PLA_2_ (P84651) |
|  |  |  |  | 1298.7 | 1 | HLLQFGDLIDK | PLA_2_ (P84651) |
|  |  |  |  | 2169.9 | 1 | SGFWYYGFYGCYCGLGGR | PLA_2_ (P84651) |
|  |  |  |  | 1505.6 | 1 | CCFVHDCCYGK | PLA_2_ (P84651) |
|  |  |  |  | 950.5 | 1 | YWLFPPK | PLA_2_ (T1DH17) |
| **84** | 15.0 | 0.345 | 5 | 2169.9 | 1 | SGFWYYGFYGCYCGLGGR | PLA_2_ (P84651) |
| **85** |  | 0.196 | 4.9 | 1241.5 | 1 | DFSWEWTDR | Gal-binding lectin (T2HP42) |
| **86** |  | 0.058 |  | - | - | - | nd |
| **87** |  | 0.190 |  | - | - | - | nd |
| **88** |  | 0.318 |  | - | - | - | nd |
| **89** |  | 0.373 |  | - | - | - | nd |
| **90** | 11.4 | 0.144 | 5.1 | 1179.6 | 1 | EYETLLKQR | Unknown protein (T2HPX7) |

LAO: L-amino acid oxidases; SVMP: Snake venom metalloproteinases; SVSP: Snake venom serine proteases; PLA_2_: Phospholipases A_2_; nd: no detected
