# Supplementary material for: Cross-reactivity, antivenomics, and neutralization of toxic activities of Lachesis venoms by polyspecific and monospecific antivenoms
Source: PLoS Negl Trop Dis. 2017 Aug 7;11(8):e0005793. doi: 10.1371/journal.pntd.0005793 (PMC5560760; doi:10.1371/journal.pntd.0005793)
Supplement: S2 Table — (DOCX) [file pntd.0005793.s003.docx]

**S2 Table.** 2DE-separated *L. stenophrys* venom protein spots recognized by mono and polyspecific antivenoms

| **Spot** | **BCL** | **BL** | **AL** | **AB** | **AC** | **Protein Family** |
| --- | --- | --- | --- | --- | --- | --- |
| 1 | + | + | + | + | + | - |
| 2 | + | + | + | + | + | LAO |
| 3 | + | + | + | + | + | LAO |
| 4 | + | + | + | + | + | LAO |
| 5 | + | + | + | + | + | - |
| 6 | + | + | + | + | + | - |
| 7 | + | + | + | + | + | - |
| 8 | + | + | + | + | + | - |
| 9 | + | + | + | + | + | - |
| 10 | + | + | + | + | + | SVMP |
| 11 | + | + | + | + | + | SVMP |
| 12 | + | + | + | + | + | SVMP |
| 13 | + | + | + | + | + | SVMP |
| 14 | + | + | + | + | + | SVMP |
| 15 | + | + | + | + | + | SVMP |
| 16 | + | + | + | + | + | - |
| 17 | + | + | + | + | + | SVMP |
| 18 | - | + | + | + | + | - |
| 19 | + | + | + | + | + | - |
| 20 | + | + | + | + | + | - |
| 21 | - | + | + | + | + | - |
| 22 | + | + | + | + | + | SVMP |
| 23 | + | + | + | + | + | SVSP |
| 24 | - | + | - | - | - | - |
| 25 | + | + | + | + | + | SVSP |
| 26 | + | + | + | + | + | - |
| 27 | + | + | + | + | + | SVSP |
| 28 | + | + | + | + | + | - |
| 29 | + | + | + | + | + | SVSP |
| 30 | - | - | - | + | - | - |
| 31 | + | + | + | + | + | SVSP |
| 32 | + | + | + | + | + | - |
| 33 | - | + | + | + | + | - |
| 34 | + | + | + | - | - | - |
| 36 | + |  |  |  |  | - |
| 34 | + | + | + | + | + | - |
| 38 | + | + | + | + | + | - |
| 39 | + | + | + | + | + | SVSP |
| 40 | + | + | + | + | + | - |
| 41 | + | + | + | + | + | SVMP |
| 42 | + | + | + | + | + | SVSP |
| 43 | + | + | + | + | + | - |
| 44 | + | + | - | - | - | - |
| 45 | + | + | + | + | + | - |
| 46 | + | + | + | + | + | SVSP |
| 47 | + | + | + | + | + | SVSP |
| 48 | + | + | + | + | + | SVSP |
| 49 | + | + | + | + | + | SVSP |
| 50 | + | + | + | + | + | SVSP |
| 51 | + | + | + | + | + | SVSP |
| 52 | + | - | - | - | - | - |
| 53 | + | + | + | + | + | SVSP |
| 54 | + | + | + | + | + | SVSP |
| 56 | + | - | - | - | - | PLA_2_ |
| 57 | + | + | + | + | + | SVSP |
| 58 | + | + | + | + | + | SVSP |
| 59 | + | + | + | + | + | SVSP |
| 60 | + | + | + | + | + | - |
| 61 | + | + | + | + | + | - |
| 62 | + | + | + | + | + | SVMP |
| 63 | + | + | + | + | + | - |
| 64 | + | + | + | + | + | SVMP |
| 65 | - | - | - | + | + | - |
| 67 | + | + | + | + | + | - |
| 68 | + | - | - | - | - | - |
| 69 | - | - | - | + | - | SVMP |
| 70 | - | - | - | + | - | - |
| 73 | + | - | + | + | + | - |
| 74 | + | - | - | + | + | - |
| 75 | + | + | + | + | + | - |
| 76 | + | + | + | + | + | - |
| 77 | - | + | - | - | - | LAO |
| 79 | + | - | - | - | - | PLA_2_, C-type lectin |
| 80 | + | - | - | - | - | C-type lectin |
| 81 |  | - | - | - | - | - |
| 82 | + | + | + | + | + | PLA_2_, Gal-binding lectin |
| 83 | - | - | + | + | + | PLA_2_ |
| 84 | - | - | + | + | + | PLA_2_ |
| 88 | - | - | + | + | + | - |

+: Recognition of spots by antivenom

(-): No recognition of spots by antivenom.

BCL: polyspecific anti-bothropic, anti-crotalic, anti-lachesic ICP antivenom; SABL: anti-bothropic and anti-lachesic antivenom from Instituto Vital Brazil; AL: monoespecific anti-lachesic antivenom; AB: monoespecific anti-bothropic antivenom; AC: monoespecific anti-crotalic antivenom.
